# αB-crystallin elastin-like polypeptides for sustained ocular drug delivery

**DOI:** 10.1007/s13346-026-02210-w

**Published:** 2026-09-02

**Authors:** Sara Aly Attia, Amanda M. Tse, Ashley D. Ramirez, Maryam Izadiar, Ying Liu, Hao Guo, Angela Albanese, Whitaker Cohn, Adrienne W. MacKay, Paul M. Seidler, Ram Kannan, Parameswaran G. Sreekumar, John Andrew MacKay

**Affiliations:** 1 Department of Pharmacology and Pharmaceutical Sciences, University of Southern California Alfred E. Mann School of Pharmacy and Pharmaceutical Sciences, Los Angeles, CA 90033, USA; 2 Department of Ophthalmology, Roski Eye Institute, University of Southern California Keck School of Medicine, Los Angeles, CA 90033, USA; 3 Titus Family Department of Clinical Pharmacy, University of Southern California Alfred E. Mann School of Pharmacy and Pharmaceutical Sciences, Los Angeles, CA 90033, USA; 4 Department of Population and Public Health Sciences, University of Southern California Keck School of Medicine, Los Angeles, CA 90033, USA; 5 Doheny Eye Institute, Pasadena, CA 91103, USA; 6 Stein Eye Institute, University of California Geffen School of Medicine, Los Angeles, CA 90095, USA; 7 Alfred E. Mann Department of Biomedical Engineering, University of Southern California Viterbi School of Engineering, Los Angeles, CA 90089, USA

**Keywords:** ER stress, ELPs, Microscopy, Apoptosis, Fibril, Proteomics

## Abstract

The unfolded protein response (UPR) has been mechanistically implicated in multiple ocular disorders, including age-related macular degeneration (AMD). Therapeutic intervention using an αB-crystallin-derived ‘mini-cry’ chaperone peptide has potential to restore proteostasis; however, its low molecular weight results in rapid ocular clearance. To prolong mini-cry’s ocular effects, our team linked it with elastin-like polypeptides (ELPs). ELPs are thermoresponsive polymers that phase-separate into microscale coacervates. Provided ELPs do not interfere with biological activity, cry-ELPs could prolong intraocular retention and improve therapeutic effect. This study compares two cry-ELPs (cry-SI and cry-V96) designed to phase-separate following intravitreal administration. First, chaperone activity was assessed using tau protein aggregation assays, demonstrating that cry-V96 exhibited superior inhibition of tau seeding and fibril disaggregation. Second, modulation of the UPR was assessed in human retinal pigment epithelium cells using tunicamycin-induced stress. Both constructs suppressed PERK/ATF-4/CHOP signaling, with cry-V96 showing the most robust attenuation of ER stress. Third, cellular uptake and subcellular localization were analyzed by biochemical fractionation and Western blotting, revealing enhanced cellular uptake and association and stress-dependent nuclear localization of cry-ELPs, compared to controls. Fourth, the lead construct, cry-V96, was evaluated in vivo in rabbit eyes, where it formed a sustained intravitreal depot, exhibited prolonged retention, and showed no evidence of ocular toxicity by fluorescence imaging and optical coherence tomography (OCT). Finally, proteomic profiling using liquid chromatography-tandem mass spectrometry (LC-MS/MS) identified 59 proteins selectively enriched by cry-V96, implicating pathways involved in proteostasis, intracellular trafficking, and stress adaptation. Collectively, these findings establish cry-ELPs as multifunctional molecules that combine potent chaperone activity with sustained ocular delivery, providing a promising strategy for targeting UPR dysregulation in AMD and related degenerative diseases.

## Introduction

AMD is a leading cause of severe vision impairment in older people and is expected to affect approximately 288 million people worldwide by 2040 [1]. This disease is a progressive disorder that affects the RPE and can lead to irreversible vision loss [2, 3]. AMD manifests in either a dry (atrophic) or wet (neovascular) form with dry AMD progressing slowly from drusen accumulation and RPE dysfunction to geographic atrophy (GA) and irreversible vision loss [4]. Currently [5, 6], Pegcetacoplan (Syfovre) and Avacincaptad Pegol (Izervay) are the only FDA-approved therapies for GA. These complement inhibitors modestly slow lesion progression (~ 14–20%) but they do not restore vision or reverse retinal damage. Clinical functional benefits remain limited [7, 8]. These limitations underscore the need for therapies that target causative disease mechanisms and preserve cellular function.

Endoplasmic reticulum (ER) stress is a key driver of retinal degeneration in multiple ocular diseases [9]. As the primary site of protein synthesis, folding, trafficking, and post-translational modifications, the ER is highly sensitive to disruptions in proteostasis [10]. ER stress signaling is mediated by three transmembrane sensors; the PKR-like endoplasmic reticulum kinase (PERK), inositol-requiring enzyme 1, and activating transcription factor 6 [11]. Under physiological conditions, glucose-regulated protein 78 (GRP78/BiP) binds to these transmembrane sensors and maintains them in an inactive state. Accumulation of misfolded proteins leads to GRP78 dissociation and activation of the UPR, an adaptive mechanism that initially restores cellular homeostasis through coordinated transcriptional and translational control [12]. However, chronic or unresolved ER stress drives apoptotic signaling and contributes to ocular pathologies, including AMD. Given the essential metabolic and functional support provided by the RPE, ER stress-induced RPE apoptosis represents a compelling therapeutic target in retinal degeneration [13].

αB-crystallin, a member of the small heat shock family, is a key molecular chaperone involved in maintaining retinal homeostasis [14]. It recognizes misfolded and aggregation-prone proteins and facilitates their folding, thereby promoting cell survival. Hinton and colleagues [15] reported that RPE cells derived from αB-crystallin knockout mice exhibited increased susceptibility to ER stress, oxidative stress, and cell death, whereas αB-crystallin overexpression reduced mitochondrial dysfunction and caspase 3 upregulation. Alge and collaborators [16] demonstrated that oxidative stress caused cell death in human RPE and overexpression of αB-crystallin provides protection. Additional studies demonstrated that αB-crystallin sequesters key proapoptotic regulators, including procaspase 3, Bax, and Bcl-X_S_, which prevents mitochondrial translocation, cytochrome c release, and downstream caspase upregulation [17]. Consistent with these findings, loss of either αA- or αB-crystallin in P23H rhodopsin mutant mice triggered ER stress, intraretinal inflammation, and accelerated photoreceptor degeneration [18].

Bhattacharyya [19] and coworkers identified a bioactive mini-cry peptide corresponding to residues 73–92 in αB-crystallin (DRFSVNLDVKHFSPEELKVK), which retains chaperone-like anti-aggregation activity comparable to intact αB-crystallin. However, both full-length αB-crystallin (~ 22 kDa) and the mini-cry (~ 2.4 kDa) are rapidly cleared following intraocular administration, limiting their therapeutic potential [20]. To address this delivery challenge, our team conjugated the mini-cry peptide to ELPs to enhance intraocular residence time and therapeutic durability [21, 22].

Derived from mammalian tropoelastin, ELPs are genetically encoded, thermoresponsive biomaterials composed of repeating pentapeptide (VPGXG) motifs. Above a transition temperature, *T_t_*, they form a liquid-in-liquid coacervate, which is governed by guest residue composition, molecular weight, and concentration. Below *T_t_*, ELPs remain soluble and nanoscale, whereas above *T*_*t*_, they self-assemble micronscale droplets. This sustains tissue retention and release within the body. In addition, they can be produced and purified from bacterial expression systems [23]. Our group previously compared the therapeutic efficacy of the mini-cry peptide with an ELP conjugate composed of two blocks, (VPGSG)_48_ and (VPGIG)_48_ (SI). Conjugation of the peptide to the amphiphilic ELP generated cry-SI, which exhibited enhanced thermoresponsive properties and prolonged intra-ocular retention compared with the free peptide, resulting in improved efficacy in a sodium iodate-induced mouse model of retinal degeneration [22, 24]. More recently, we developed cry-V96 by conjugating the mini-cry peptide to a (VPGVG)_96_ backbone. cry-V96 demonstrated efficient lysosomal escape while maintaining the cytoprotective activity of cry-SI in oxidatively stressed ARPE-19 cells [22]. Collectively, these findings support the development of ELP-based sustained ocular therapeutics and provide the rationale for the αB-crystallin ELP constructs evaluated in the present study.

In our previous study, cry-SI and cry-V96 demonstrated chaperone activity using conventional aggregation assays, including insulin and alcohol dehydrogenase models [21, 22]. However, those assays offer limited insight into how engineered chaperone peptides function under disease-relevant, intracellular conditions. Here, for the first time, we report that exogenous cry-ELP fusions can disaggregate intracellular tau as a model of chaperone activity, protect RPE cells from tunicamycin-induced ER stress, and form a long-lasting depot after intravitreal injection to the rabbit eye. Together, these results suggest cry-V96 is a promising, long-acting chaperone for retinal degenerative diseases driven by the UPR.

## Methods

### ELP preparation and characterization

cry-ELP conjugates were expressed in ClearColi bacteria and purified in phosphate buffered saline (PBS, Genesee Scientific, El Cajon, CA, USA #25–508) as reported [21, 22, 24, 25]. Sodium dodecyl sulfate-polyacrylamide gel electrophoresis (SDS-PAGE) was used to characterize purity of four constructs (SI, cry-SI, V96, and cry-V96) following ELP-mediated purification. ELPs (0.6 mg/mL) were mixed with 2-mercaptoethanol (Thermo Fisher Scientific, Waltham, MA, USA #31350010) in Laemmli buffer (Bio-Rad Laboratories, Hercules, CA, USA #1610747) and heated at 95 °C for 5 min. Denatured samples and prestained protein ladder (Bio-Rad Laboratories #1610375) were loaded onto a 4–20% SDS-PAGE gel (Bio-Rad Laboratories #4561095, #1610732). The gel was soaked for 10 min in prestain buffer (0.19 M Trizma base from MilliporeSigma, Burlington, MA, USA #T1503 and 0.1% SDS (Thermo Fisher Scientific #15525017), incubated for 3 min in 0.3 M of Copper (II) chloride dihydrate (Fisher Scientific, Pittsburgh, PA, USA #AC206345000), and imaged using the ChemiDoc Touch system (Bio-Rad Laboratories).

Thermosensitivity of ELP constructs was evaluated in PBS using a Cary 3500 Multicell UV-Vis spectrophotometer (Agilent Technologies, Santa Clara, CA, USA). Briefly, 1 mL of each construct (10 μM) was loaded into polystyrene cuvettes (Agilent Technologies, #6610018700) and sealed with parafilm. Samples were heated from 15 to 50 °C and cooled from 50 to 15 °C at 0.5 °C/min. The spectrophotometer was blanked and absorbance at 350 nm was recorded. The maximum first derivative of each curve was defined as *T_t_*. The data were compared with previously collected data from a different instrument (Beckman Coulter, Brea, CA, USA) [22] to evaluate whether the experimental results aligned with the predictions. Data were exported and plotted using GraphPad Prism software.

### Preparation of Alzheimer’s disease (AD) crude brain homogenate and purified AD tau fibrils

De-identified human autopsy samples from the UCLA Pathology Department were obtained from AD donors who consented to autopsy (Clinical trial number: not applicable). Sections of ~250 mg of autopsy brain tissues were cut and homogenized using a Kinematica Polytron (Kinematica AG, Lucerne, Switzerland #PT 10–35) in 750 μL sucrose buffer composed of 0.8 M sodium chloride (NaCl, VWR, Radnor, PA, USA #97063-368), 10% sucrose (VWR #97063-790), 10 mM Tris(hydroxymethyl)aminomethane, pH 7.4 (Fisher Scientific #T395), supplemented with 1 mM ethyleneglycol bis(2-aminoethyl ether)-N, N,N’,N’-tetraacetic acid (EGTA, MilliporeSigma #324626) and 5 mM ethylenedi-aminetetraacetic acid (EDTA, MilliporeSigma #E9884). Homogenates were flash frozen at −80 °C and later used as tau seeds for cell culture assays.

To obtain purified tau fibrils, ~2–6 g of autopsy brain tissue was homogenized in 10 mL sucrose buffer. Homogenates were centrifuged at 20,100x g for 20 min at 4 °C, and the supernatants were collected and transferred to airfuge ultra tubes and spun at 95,000 RPM for 60 min (Beckman Coulter). The resulting pellet was resuspended in 2.5 mL sucrose buffer supplemented with EDTA (5 mM) and EGTA (1 mM) and centrifuged at 20,100x g for 30 min at 4 °C. The supernatant was spun at 95,000 RPM for 60 min at 4 °C, the pellet containing tau fibrils was resuspended in 100 μL of 20 mM Tris-HCl (pH 7.4) containing 100 mM NaCl.

### HEK293T tau biosensor seeding assay

HEK293T tau K18-CY biosensor cells were obtained from Marc Diamond’s laboratory (University of Texas South-western Medical Center). They stably express the K18 aggregation-prone tau fragment spanning residues 244 to 372, which have P301S and V337M point mutations, that is fused to YFP [26]. Cells were cultured in Dulbecco’s Modified Eagle Medium (Thermo Fisher Scientific #11965092) supplemented with 10% Fetal Bovine Serum (FBS, Thermo Fisher Scientific #A3160401), 1% penicillin/streptomycin (Thermo Fisher Scientific #15140122), and 1% Gluta-MAX (Thermo Fisher Scientific #35050061) in a T25 flask at 37 °C with 5% CO_2_. Biosensor cells were plated in a 96-well plate (USA Scientific, Ocala, FL, USA #CC7682-7596) at about 40–60% confluence in a volume of 100 μL 24 h before transfection.

Treatment groups (SI, cry-SI, V96, and cry-V96) of different concentrations (1, 5, 10, 25 μM) were preincubated with AD crude brain homogenate at a ratio of 1 μL homogenate diluted in 19 μL Opti-MEM (Thermo Fisher Scientific #31985062) at 4 °C overnight prior to transfection. Homogenates were sonicated for 3 min at 40% power to enhance seeding. For transfection, Lipofectamine 2000 (Thermo Fisher Scientific #11668019) was used [27]. Non-transfected cells served as blank control, while cells transfected with brain homogenate alone served as positive control for tau seeding. Fluorescent images were acquired using a BioTek Cytation5 microscope (Agilent Technologies) with a 4x objective from the seeded 96-well plates. Images were captured in the GFP channel using a 3x2 montage to maximize coverage of the seeded area in each well. Acquired images were stitched, preprocessed, and deconvolved for high-content image analysis. Image analysis was performed using ImageJ software (version 1.54f) to quantify tau puncta per well [28]. Fluorescence from unseeded cells was subtracted, and aggregates were counted as peaks above background using the particle analyzer plugin [29]. Puncta counts were normalized to the cell confluence determined by image analysis. For dose-dependent experiments, each concentration was tested in triplicate, and the average number of puncta was calculated. Statistical analyses were performed using GraphPad Prism software.

### Preparation of negative stain grids

Sarkosyl-insoluble tau fibrils derived from autopsy AD brain tissue were used for transmission electron microscopy (TEM) analysis. These purified AD tau fibrils were diluted 1:10 in 1× PBS for negative stain grids. 6 μl of diluted samples were pipetted onto a carbon-coated copper grid (Electron Microscopy Sciences, Hatfield, PA, USA #FCF-150-Cu) and incubated at room temperature for 3 min. Excess liquid was removed by blotting the edge of the grid with filter paper. The grid was then immediately treated with 10 μl of negative stain solution (4% uranyl acetate) for another 2 min and excess stain was blotted off. The grid was allowed to air dry perpendicularly for 10 min before being stored in a grid box for further analysis. To obtain images (VWR, Electron Microscopy Sciences), negative-stain grids of each sample were screened at a magnification of ×12,000 on a JEOL 2100 TEM, and images were collected in 5-micron increments. Fibrils were counted from collections of 50 micrographs for each experimental condition, including purified fibrils treated with 25 μM of control ELPs incubated at room temperature for 24 h.

### Western blotting

ARPE-19 (American Type Culture Collection, Manassas, VA, USA #CRL-2302) and primary human RPE cells (5 · 10^5^) were seeded in 6-well plates (Bioland Scientific LLC, Paramount, CA, USA #703003), grown to 60% confluence, and serum-starved overnight in medium containing 1% FBS (Corning, Corning, NY, USA #35-011-CV). The primary RPE cells were isolated from human fetal eyes received from Advanced Bioscience Resources (Alameda, CA, USA) and cultured as previously described [30]. hRPE cells between passages 2 and 4 were used in all experiments. Cells were treated with ELP formulations (25 μM) for 24 h and subsequently challenged with tunicamycin (Thermo Fisher Scientific #J67401.XF) for 24 h. Following tunicamycin stress, cells were washed with cold PBS containing calcium and magnesium (Corning #21-030-CV), and lysed on ice for 1 h in RIPA buffer (Thermo Fisher Scientific #89900) with protease/phosphatase inhibitor cocktail (Cell Signaling Technology, Danvers, MA, USA #5872), vortexed for 1 min every 10 min. Lysates were clarified by centrifugation at 14,000 · g for 20 min at 4 °C. Protein concentration was measured using the Pierce BCA protein assay kit (Thermo Fisher Scientific #23225).

Equal amounts of protein (30–50 μg) were loaded onto 4–20% SDS-PAGE gels. Proteins were separated and transferred onto nitrocellulose membranes (Thermo Fisher Scientific #IB23001) using iBlot2 Dry Blotting System (Fisher Scientific #IB21001). Membranes were blocked in 5% bovine serum albumin (MilliporeSigma #I6634) for 1 h and incubated overnight at 4 °C with primary antibodies against ATF-4, CHOP, and β-actin (Cell Signaling Technology #11815, #5554, #3700; 1:1000 dilution). After washing, membranes were incubated with HRP-conjugated secondary antibodies (1:3000; Cell Signaling Technology #7074 and #7076). Signals were detected using ECL substrate (Thermo Fisher Scientific #32106) and quantified in ImageJ software as follows;

(1) $$Signal\;quantification=\frac{I_{\;\text{protein}\;\text{of}\;\text{interest}}-I_{\text{background}}}{I_{GAPDH}-I_{\text{background}}}$$

*I* refers to band integrated intensity within a consistent region of interest. β-actin was used as the loading control.

The detected bands corresponded near the expected molecular weights, compared to the protein ladder (#1610375), according to the manufacturer’s protocols. While CHOP is expected to migrate at ~27 kDa, a band near 37 kDa reference band was observed under non-stressed conditions. Upon stress induction, an additional band appeared at ~ 27 kDa. Both bands were quantified in the study [31].

### Cellular association assays

For nuclear-cytosol fractionation, ARPE-19 cells (5 x 10^5^) were seeded in 6-well plates. Confluent cells were serum-starved for 24 h, treated with ELP formulations (25 μM), and incubated with tunicamycin (0.1 μg/mL) for 24 h. Cells were trypsinized (Thermo Fisher Scientific #25300054) and separated into nuclear and cytosolic fractions following the manufacturer’s instructions (Abcam, Cambridge, MA, USA #ab289882). Protein concentration was measured using the BCA protein assay kit. Western blots were probed with an anti-ELP antibody (mouse ELP AK-1; 1:2000, Cancer Therapeutics Laboratories) [32], followed by a goat anti-mouse IgM HRP-linked secondary antibody; 1:2000, Fortis Life Sciences, Waltham, MA, USA #A90-101P). HDAC2 and GAPDH (Cell Signaling Technology #2540, #97166, #7074, and #7076) were used as cytosolic and nuclear markers. They were imaged simultaneously using a Chemi-Doc Touch system. The detected bands corresponded to the expected molecular weights, compared to the protein ladder (#1610375), according to the manufacturer’s protocols.

For microscopic assay, ARPE-19 or hRPE cells (5 × 10^3^) were seeded in a 96-well black, clear-bottom plates (Thermo Fisher Scientific #152037). After reaching 60% confluence, cells were serum-starved and treated with ELP and tunica-mycin- formulations as described above. Cells were then fixed with 4% paraformaldehyde (Thermo Fisher Scientific #043368), permeabilized by 0.1% Triton (MilliporeSigma #T8787), stained with Hoechst (Thermo Fisher Scientific #62249), blocked, stained for ELPs as previously detailed [22], and imaged using a 20× objective on a confocal fluorescence microscope (Zeiss, Thornwood, NJ, USA). Background-subtracted integrated density from the most two fluorescent stacks was quantified using Fiji software (version 2.16.0/1.54p) and then averaged for comparison.

### In vivo pharmacokinetic study

New Zealand Pigmented (NZP) female rabbits (~3 kg, 13 weeks old) were obtained from Western Oregon Rabbit Company (Philomath, OR, USA) and used to assess the ocular pharmacokinetics of fluorescein-labeled cry-V96 following a single intravitreal injection (300 μM ELPs, 120 μM fluorescein in 50 μL). Fluorescein labeling (Thermo Fisher Scientific #46410) and quantification [33] yielded a final fluorescein concentration of 120 μM, corresponding to ~ 40% labeling efficiency. Prior to injection, the formulation was filtered through an Acrodisc Mustang E membrane filter (Cytiva Life Sciences, Marlborough, MA, USA #MSTG25E3), treated with polymyxin B sulfate (MilliporeSigma #P1004), and tested for endotoxin using a HEK-Blue assay (InvivoGen, San Diego, CA, USA #rep-lps2). Only preparations with endotoxin levels below 0.2 EU/mL were used [34]. cry-V96 and PBS control (50 μl each) were injected into the left and right eyes, respectively, using a 30-guage beveled needle.

Rabbits were anaesthetized with intramuscular ketamine (25 mg/kg) and midazolam (2.5 mg/kg), with supplemental doses (25–50% of the initial dose) given as needed. The ocular surface was disinfected with povidone-iodine, followed by application of sterile saline, and 0.5% proparacaine for local anesthesia. Pupils were dilated with 2.5% phenylephrine hydrochloride and 1% tropicamide prior to fundus imaging. Aqueous humor samples were collected and OCT was performed using Spectralis OCT (Heidelberg Engineering, Dossenheim, Germany) over the 29-day study. During anesthesia, rabbits were maintained on a heated, water-circulated blanket and vital signs (heart rate, respiratory rate, and body temperature) were monitored every 5 min. After the procedure, topical neomycin/polymyxin B sulfate and bacitracin ointments were applied. Anesthesia was reversed with flumazenil (0.05 mg/kg intramuscular), and meloxicam (0.6 mg/kg, subcutaneous) was administered for analgesia. Rabbits were euthanized on day 29, and vitreous samples were collected. All materials were provided and procedures conducted in accordance with guidelines approved by the Institutional Animal Care and Use Committee at the University of Southern California.

Fluorescent tracer in aqueous humor samples was quantified using an H1 Hybrid Multi-Mode Reader (BioTek, Winooski, VT, USA) against a standard curve of purified fluorescent cry-V96. MRT was calculated as follows [35]:

(2) $$MRT=\frac{AUMC}{AUC}$$

where AUMC and AUC represent the areas under the first-moment and concentration-time curves, respectively. Vitreous humor fluorescence was quantified only in the conclusion of the study; furthermore, all samples were quantified using a plate reader. Background fluorescence was similarly assessed in samples obtained from PBS-injected eyes.

To confirm cry-V96 purity and stability throughout the study, 0.2 mg/mL fluorescent cry-V96 was run alongside aqueous humor samples on SDS-PAGE. Samples were electrophoresed, imaged with the iBright FL1000 system (Thermo Fisher Scientific), stained with Coomassie G-250 (Bio-Rad, #1610786), and imaged using the ChemiDoc Touch system.

### ELP-mediated proteomics of crystallin-binding proteins

hRPE cells stressed with 0.1 μg/mL tunicamycin were lysed in T-PER tissue protein extraction reagent (Thermo Fisher Scientific #78510), supplemented with 1× protease/phosphatase inhibitor cocktail. Lysates were mixed with V96 and cry-V96 to a final ELP concentration of 150 μM and incubated for 2 h at 4 °C with gentle shaking. ELP phase separation was induced by incubation in a 37 °C water bath, followed by three cycles of hot centrifugation, and the resulting pellets were resuspended in PBS. At 150 μM [22], *T_t_* of V96 and cry-V96 are 28 °C and 26 °C, respectively. Thus, both constructs are expected to undergo coacervation at 37 °C.

Samples were further prepared in lysis buffer with the following composition: 12 mM sodium lauroyl sarcosinate, 0.5% sodium deoxycholate, 50 mM triethylammonium bicarbonate (TEAB), and Halt protease and phosphatase inhibitor cocktail (Thermo Fisher Scientific #78446). Protein concentration was determined using a BCA protein assay. An aliquot of each sample (10 μg) was reduced and alkylated with 10 mM Tris(2-carboxyethyl)phosphine and 40 mM chloroacetamide for 20 min at 95 °C. For buffer exchange, SP3 SpeedBead magnetic carboxylate-modified particles (250 μg total, Cytiva #65152105050250 and #4515210505250, mixed 1:1) were added. Ethanol was added to a final concentration of 55% and samples were gently agitated for 15 min to facilitate protein binding, followed by three washes with 80% ethanol. Proteins were eluted from the beads with 50 μL of 50 mM TEAB, spiked with 0.5 μg of mass spectrometry-grade Trypsin/Lys-C mix (Promega, Madison, WI, USA #V50711), and digested overnight at 37 °C for 16 h.

LC-MS/MS was performed to analyze peptide samples. A 200 ng peptide aliquot was injected onto a μPAC Neo Plus C18 column (50 cm; Thermo Fisher Scientific #COL-NANO050NEOB) equilibrated in solvent A (0.1% formic acid) and separated using a Vanquish Neo UPLC system (Thermo Fisher Scientific) with an optimized 28 min gradient of solvent B (acetonitrile/water/formic acid, 80/20/0.1, v/v/v) as follows (min/%B/flow rate μL/min): 0/4/0.75, 4.4/10/0.3, 13.1/20/0.3, 20/35/0.3, 20.5/55/0.3, 20.7/99/0.3, 26/99/0.3, 26.2/99/0.75, 28/99/0.75. Eluted peptides were introduced into a nanospray ionization source coupled to an Orbitrap Astral mass spectrometer (Thermo Fisher Scientific) operated in data-independent acquisition mode. Full MS scans were acquired in the Orbitrap (m/z 380–980; AGC target 500%; maximum injection time 3 ms; resolution 240,000 at m/z 200). MS2 scans were acquired in the Astral analyzer using 2 m/z isolation windows, 25% HCD collision energy, AGC target 300%, maximum injection time 3 ms, and a resolution of 17,500 at m/z 200. Raw proteomic data were searched against a custom database encompassing the complete human proteome and common mass spectrometry contaminants using CHIMERYS in Proteome Discoverer (version 3.3, Thermo Fisher Scientific). Static modifications included carbamidomethyl (+ 57.021) on cysteine (C) and dynamic modifications included oxidation (+ 15.995). Detection of b- and y- fragment ions from tryptic peptides was utilized to identify and quantify high-confidence peptides (FDR < 1%) belonging to proteins within the provided database. Normalization was not performed because samples were enriched by association with either cry-V96 or V96. The proteomics analysis identified high-confidence protein groups, highlighting the robustness of sample preparation and the depth of the analytical platform, which enabled extensive proteome coverage. Of thousands of identified targets, 59 proteins were considered hits if identified in at least 3 of 4 replicates with at least 10-fold greater abundance by cry-V96 relative to controls treated with V96 (adjusted *p* value < 0.05; Supplementary Table S1). These hits are reported as putative αB-crystallin (CRYAB/HspB5)-interacting partners, of which a subset of these were validated by prior published studies. Proteomics data were submitted to ProteomeXchange via the PRIDE database (Project accession: PXD081775).

### Statistical analysis

Student’s *t*-test was used to assess statistical significance between two groups, while one-way ANOVA followed by Tukey’s post hoc test was used for comparisons among three or more groups. Two-way ANOVA followed by Sidak’s post hoc test was used to compare two independent variables. Where indicated, log-transformed data were used to meet homogeneity of variance. Data were analyzed using Graph-Pad Prism software (versions 9.2.0 and 9.5.1).

## Results

### ELPs exhibit reproducible purification and thermoresponsive behavior

ELP constructs were genetically engineered and stored in 25% glycerol using ClearColi BL21 to ensure consistent protein production [22, 36]. Purity and molecular weight were confirmed by SDS-PAGE with Copper(II) chloride staining (Fig. 1A). Thermosensitivity of ELPs was evaluated by measuring the optical density (OD) during heating and cooling (Fig. 1B). Only cry-SI showed irreversible coacervation at the temperatures assessed. Consistent with prior observations [22], SI does not undergo bulk phase separation at these temperatures. In contrast, both cry-V96 and V96 phase-separate reversibly close to physiological temperature.

### cry-ELPs disaggregate mutant tau in a biosensor cell model

To investigate the chaperone potential of cry-ELPs, a HEK293T tau biosensor cell model was used to detect amyloid formation. In neurodegenerative diseases like AD, accumulation of intraneuronal tau fibrils due to excessive protein aggregation is a pathological hallmark. Tau aggregation is driven by prion-like seeding (Fig. 2), whereby pathological fibrils template aggregation of monomers into new fibrils (Fig. 3) [37, 38]. Human AD brain homogenates were preincubated with various concentrations (1–25 μM) of ELPs and transfected into cells expressing aggregation-prone YFP-fused tau [39]. Seeded aggregation produces bright green puncta [40], representing intracellular tau aggregates templated by AD-derived fibrils (Fig. 2A–F, white arrows). SI and cry-SI increased seeding in a dose-dependent manner up to 10 μM, but cry-SI inhibited seeding by about 50% at 25 μM (Fig. 2G). In contrast, cry-V96 inhibited seeding in a dose-dependent manner, achieving near-complete suppression at 25 μM, whereas V96 showed no effect. Area under the curve (AUC) analysis over this concentration gradient revealed a significant difference only between V96 and cry-V96 (Fig. 2H). These results suggest that cry-ELPs, particularly cry-V96, inhibit tau seeding, possibly by disaggregating tau and eliminating seeding templates.

### cry-ELPs promote tau fibril disaggregation

To investigate the mechanism by which cry-ELPs inhibit seeding in biosensor cells, their ability to disaggregate tau fibrils was assessed. AD tau fibrils purified from autopsied human brain were pretreated with 25 μM of SI, cry-SI, V96 or cry-V96 for 24 h at room temperature. Fibrils were deposited onto negatively stained TEM grids and manually counted (Fig. 3A–E). cry-ELPs reduced fibril counts by about 50%, compared to control ELPs (Fig. 3F), suggesting disaggregation activity. These data demonstrate that crystallin maintains its chaperone activity and, more specifically, that tau disaggregation occurs when crystallin is fused to ELPs. While the treatment in HEK293T-tau seeding assay was performed at 4 °C (Fig. 2), fibril disaggregation was conducted at room temperature. cry-ELPs preserved their activity under both temperatures.

### cry-ELPs attenuate ATF-4 upregulation under tunicamycin-induced ER stress

Having established the robust chaperone activity of cry-V96 in a model of tau aggregation, we next sought to determine whether cry-ELPs modulate ER stress signaling through the UPR. Specifically, ATF-4 activation was studied in ARPE-19 cells as a functional readout of the PERK arm of the UPR. ATF-4 is a central transcriptional effector that integrates PERK activation with downstream adaptive and apoptotic gene expression programs [9]. Prior to evaluating effects of cry-ELPs on UPR signaling, a WST-1 viability assay was performed to determine the cellular response to tunicamycin (0.01, 0.1, 1.0, and 10 μg/mL, Supplementary Fig. S1). Cell viability was 80 ± 3, 76 ± 6, 60 ± 2, and 65 ± 3%, respectively. To assess ATF-4 regulation, serum-starved ARPE-19 cells were challenged with different concentrations of tunicamycin (Fig. 4A). The ELP concentration was fixed at 25 μM to ensure coacervation under physiological temperature [22]. ATF-4 levels were normalized to β-actin (Fig. 4B), and the AUC across all tunicamycin concentrations was compared (Fig. 4C). ATF-4 upregulation remained highest in the control SI and V96 groups, indicating robust PERK pathway activation under ER stress. In contrast, both cry-SI and cry-V96 significantly reduced ATF-4 across the range of tunicamycin concentrations. Collectively, these data support cry-ELPs as modulators of stress-responsive signaling network, linking their chaperone function to suppression of maladaptive UPR activation.

### cry-ELPs exhibit enhanced cellular uptake and nuclear localization under tunicamycin-induced stress

Cellular uptake and subcellular localization of αB-crystallin, particularly its nuclear translocation, have been associated with cellular stress responses and are thought to contribute to its cytoprotective functions, although a direct requirement for nuclear localization in ER stress protection remains to be fully established [41]. Accordingly, we examined the cellular association and intracellular distribution of cry-ELP formulations, with a focus on cytosolic and nuclear compartments. A biochemical nuclear/cytosolic fractionation method was employed [42], followed by anti-ELP Western blotting under basal conditions and tunicamycin-induced ER stress (Fig. 5A, B). Fractionation revealed that cry-ELPs were significantly more abundant than control ELPs in both nuclear and cytosolic fractions, and their distribution was altered under tunicamycin challenge. When normalized to the nuclear HDAC2, nuclear accumulation of cry-ELPs increased under ER stress. In contrast, normalization to cytosolic GAPDH indicated a relative reduction in cytosolic abundance, consistent with stress-dependent redistribution to the nucleus. Fluorescence microscopy confirmed these findings, demonstrating markedly higher uptake and intracellular retention compared to control ELPs under stress conditions (Fig. 5C, D). Collectively, these results demonstrate that tunicamycin-induced ER stress enhances both cellular uptake and nuclear localization of cry-ELPs.

### cry-ELPs maintain cellular protection and association under tunicamycin-induced ER stress

Since cry-V96 showed superior depot reversibility (Fig. 1), chaperone activity (Figs. 2 and 3), and comparable protection against tunicamycin (0.1 μg/mL) (Fig. 4), subsequent mechanistic studies focused on the protective potential of cry-V96. After establishing its effects in ARPE-19 cells, this evaluation was extended to hRPE cells, which more closely recapitulate native RPE physiology than ARPE-19 [44]. ATF-4 (Fig. 6A–B) and CHOP (Fig. 6C–D) upregulation were studied by Western blot following tunicamycin challenge in the presence of cry-V96 or controls. Prior to these assays, WST-1 analysis showed 75 ± 4% cell viability under tunicamycin-stress (Supplementary Fig. S1). ATF-4 protein was used as a proximal readout of PERK-eIF2α pathway activation, whereas CHOP, a direct downstream effector of ATF-4 and key modulator of ER-stress induced apoptosis, was assessed to determine downstream pro-apoptotic signaling outcomes [9]. In hRPE, cry-V96 significantly attenuated tunicamycin-induced upregulation of ATF-4 and CHOP, indicating suppression of the PERK-UPR signaling and its downstream apoptotic transcriptional program. Confocal microscopy (Fig. 6E–F) further confirmed enhanced cellular association of cry-ELPs under ER stress conditions, consistent with findings in ARPE-19 cells (Fig. 5). Collectively, these results demonstrate that cry-ELPs suppress ER stress-induced PERK-ATF-4-CHOP signaling, while simultaneously increasing cellular association under stress.

### cry-V96 forms a sustained depot in the vitreous humor

Following demonstration of cry-ELPs protection using in vitro RPE models (Figs. 4 and 6), the ocular pharmacokinetics of cry-V96 were evaluated in NZP rabbits, a validated surrogate model for human ocular studies due to its comparable ocular size and melanin distribution [45]. cry-V96 was labeled using NHS-carboxyfluorescein chemistry at primary amines, yielding a labeling ratio of 0.4 fluorophores per molecule. The labeled formulation was administered intravitreally and monitored longitudinally by Spectralis OCT (Fig. 7A) [46]. This enabled non-invasive visualization of vitreous distribution over time, with infrared reflectance (IR) mode providing structural imaging and fluorescein angiography (FA) mode enabling detection of labeled cry-V96. PBS-injected eyes showed no FA signal, confirming the absence of non-specific fluorescence or non-specific tissue interaction (Supplementary Fig. S2). OCT imaging demonstrated a sustained cry-V96 depot within the vitreous.

In aqueous humor samples (Fig. 7B, Supplementary Fig. S3), cry-V96 reached peak levels at day 1, after which it exhibited a terminal half-life of 13 ± 4 days. Its MRT was 64 ± 28 days (Eq. 2) and the apparent clearance from the aqueous humor (CL/F) was 0.74 ± 0.43 mL/day. At 29 days post-injection, the vitreous concentrations were 696 ± 485 nM, corresponding to 15 ± 9% (*n* = 3) of the injected dose. To augment these measures, additional eye injections were obtained at 0 and 8 days after independent administration of cry-V96. At days 8 and 0, 24 ± 6% (*n* = 4) and 71 ± 15% (*n* = 3) of the injected dose were recovered from enucleated vitreous. When combined with day 29 data, together these estimated a 13.6 day half-life, which was consistent with the half-life of the aqueous humor sampling. Importantly, longitudinal OCT showed no evidence of retinal inflammation, edema, or structural disruption following cry-V96 administration (Fig. 7C, D), indicating favorable ocular tolerability. While OCT imaging provides real-time visualization of the depot, aqueous humor sampling reflects discrete compartmental measurements. Together, these measures provide complementary pharmacokinetic information. Finally, stability of cry-V96 in the vitreous was evaluated using SDS-PAGE coupled with fluorescence detection. In two of three animals, this signal remained detectable. There, cry-V96 migrated at the expected molecular weight of the intact formulation (Fig. 7E), indicating minimal degradation over the study period. No fluorescence signal was detected in the PBS-injected control eyes. These studies demonstrate extended release from a cry-V96 depot, which may justify dosing once every few months.

### Proteomic profiling identifies proteins enriched by cry-V96

The ELP-mediated phase separation is useful for enriching ELP-linked fusion proteins [22]; however, the interactome of these fusions remains largely unknown. This was especially true for cry-V96, which is functionalized with a crystallin-derived mini-chaperone peptide. Accordingly, cry-V96 was expected to interact with multiple proteins, a subset of which may mediate cellular internalization, endolysosomal escape, and protection against cellular stress.

To identify these interactions, we designed a novel proteomics-based approach to capture proteins potentially involved in cry-V96-mediated protection of RPE under stress. Cultured hRPE were challenged by tunicamycin, lysed, and the resulting lysates were incubated with either cry-V96 or V96. Experiments were performed in quadruplicate and samples were enriched through three rounds of ELP-mediated purification. Enriched fractions were subjected to deep proteomic profiling, which identified 4,848 unique proteins in the V96 coacervate.

For cry-V96, 59 proteins were reproducibly enriched (detected in at least three replicates with greater abundance relative to V96). Functional annotation was performed using Enrichr [47] (Supplementary Table S1). Gene Ontology (GO) analysis, including Cellular Component, Compartment Analysis, as well as Jensen DISEASES (Curated 2025), revealed that approximately 27 of the cry-V96-enriched proteins were nuclear, with the ER representing the most significantly enriched compartment. Disease association analysis identified eye and nervous system diseases as top-ranked categories. To further prioritize candidates, proteins were filtered based on reported associations in peer-reviewed literature using ChatGPT.edu (GPT-5.3) [48], with endpoints assessed in this study. This resulted in a refined list of 20 proteins, which were grouped according to putative functional categories (Table 1). Of these proteins, TUBB, CHMP4B, PTGES3, and A2M have been reported in the literature to interact with other Hsp, including αB-crystallin. Lastly, we employed fluorescence anisotropy to estimate the binding constant of cry-V96 to three proteins on this list, including UBE2H, SUB1, and CHMP4B (Supplemental Fig. S5). Thus, in addition to identifying novel interactions cry-V96, this screen successfully identified proteins known to interact with chaperone proteins.

## Discussion

Restoration of intracellular protein homeostasis represents a promising therapeutic direction for treating AMD. Molecular chaperones, particularly αB-crystallin, play important roles in maintaining retinal proteostasis by preventing aggregation of misfolded proteins and stabilizing stress-sensitive cellular proteins. However, αB-crystallin expression declines with aging and chronic oxidative stress, leading to increased vulnerability of cells to ER stress and protein aggregation. This decline is particularly relevant in AMD, where chronic metabolic stress, lipid accumulation, and inflammation converge to overwhelm cellular quality control systems [80, 81]. In a proteomic study from [82] Nakata and collaborators, they revealed elevated levels of both αA- and αB-crystallins in Bruch’s membrane and choroidal connective tissues, structural and mechanical support for retina [83], in AMD donor eyes, which likely reflects a disease-related response and defensive role. Sreekumar and collaborators [84] reported that RPE cells produce αB-crystallin through exosomal secretion, and its exogenous administration is more taken up into the nucleus and cytosol of neighboring cells, where it exerts anti-apoptotic effect. These findings consistently align with our observations of increased nuclear and cytosolic uptake for only crystallin-mediated ELPs, as investigated by subcellular fractionation followed by Western blotting (Fig. 5A–B) and confocal microscopy (Figs. 5C and 6E). Our collaborators [85] additionally demonstrated that αB-crystallin-knockout mice exhibited increased susceptibility to epithelial-mesenchymal transition of RPE cells, a process contributing to subretinal fibrosis observed in late-stage neovascular AMD. This influences the role of αB-crystallin in modulating RPE response and slowing AMD progression. A more recent study by the Kannan group [86] concluded that senescent RPE cells under stress drive mitochondrial dysfunction, thus contributing to AMD. They administered free mini-cry peptide in the following experimental models: (i) doxorubicin and H_2_O_2_-induced senescent RPE cell culture models, (ii) NaIO_3_-induced retinal degeneration mice model, and (iii) subretinal fibrosis mice model induced by laser-induced choroidal neovascularization. Over these studies, senescence and pro-inflammatory markers were suppressed, while mitochondrial function was restored. Our study is based on this biological rationale by engineering this mini-cry peptide to ELPs to enhance stability, intracellular delivery, and functional chaperone activity.

Our previous studies established that αB crystallin-derived mini-cry and ELP fusions retain chaperone activity in classical aggregation systems (e.g., insulin-DTT-induced and alcohol dehydrogenase assays) [21, 22]. Here, we extend these findings to more disease-relevant models of tau aggregation and ER stress-induced proteotoxicity. cry-V96 demonstrated strong anti-aggregation activity, significantly reducing tau fibril formation and seeding capacity in biosensor assays (Fig. 2), and decreasing fibrillar structures observed by electron microscopy (Fig. 3). These findings indicate that cry-V96 retains functional chaperone activity when fused to ELPs. Importantly, these data reveal that ELP architecture critically influences biological activity. While cry-V96 consistently suppressed aggregation, the amphiphilic ELP called SI showed concentration-dependent enhancement of aggregation, highlighting the importance of ELP design in modulating therapeutic peptides [87].

With regard to the biology of crystallins, Hampton and coworkers [88] demonstrated that intracerebral administration of recombinant human αB-crystallin in P301S tau mutant mice enhanced microglia and astrocyte-derived neurotropic signaling, thereby supporting neuronal survival in cortical regions. Conversely, Bai and coworkers [89] reported elevated tau accumulation in brains of aged αA, αB-, and double αA/αB-crystallin knockout mice, suggesting a regulatory relationship between crystallins and tau proteostasis. Other heat shock proteins (Hsps) also regulate tau aggregation [90–92]. These observations place αB-crystallin within the broader network of chaperone-mediated tau regulation. Chaperone systems act at multiple stages of tau misfolding and aggregation, and their function may be engineered as protein therapeutics. Interestingly, emerging evidence suggests overlap between retinal and brain proteinopathies, as demonstrated by the detection of pathological p-tau in both retina and postmortem AD brain tissue [93]. Ocular diseases such as cataracts, which are associated with amyloid fibril deposits, also may provide a platform to evaluate αB-crystallin-ELP formulations [94].

To the best of our knowledge, this report is the first use of ELP-mediated assembly to identify targets of a fused peptide, like mini-cry, using unbiased proteomics. After subtracting proteins captured by unmodified V96, this identified proteins that associate directly or indirectly with the cry-V96 (Table 1). While future studies are required to validate and understand their implications, many of those targets have biological effects possibly related to this model. We break these into three categories: (i) protection against UPR; (ii) relevance to AD; and (iii) cellular trafficking.

As these hits relate to UPR, several classes of proteins that bind cry-V96 play a role in ocular disease. First enrichment of ESCRT components (CHMP4B, CHMP4A, and CHMP5) suggests involvement in membrane remodeling, endolysosomal trafficking, and cytosolic delivery [50]. CHMP4B, enriched ~ 48-fold, has also been implicated in ER stress recovery *via* micro-ER-phagy [51] and recruitment to damaged endolysosomal membranes [95]. Enrichment of translation initiation-related proteins (EIF1AD, EIF1AX, EIF1AY) suggest modulation of stress-adaptive translation and redox homeostasis [56, 57, 96]. Ross and collaborators [59] reported that EIF1A promoted uORF2-mediated-down-regulation of ATF-4 translation. In agreement (Figs. 4 and 6A–D), our Western blot for both ATF-4 and its downstream target (CHOP) were downregulated only in cry-ELP treated groups. A third, enriched protein with relevance to UPR is RCN1, an ER calcium-binding protein. RCN1 modulates the PERK-CHOP pathway [63]. Lastly, ANP32B has been implicated in cell survival through inhibition of caspase 3, a key executioner of apoptosis [64]. We previously [22] demonstrated that cry-V96 inhibited caspase 3. The enrichment of multiple proteins by cry-V96 that interact with the UPR pathway raises the possibility one or more of these have a mechanistic role in mitigation of ER stress.

As these hits relate to AD, two members of the tubulin family involved in microtubule structure and dynamics were enriched by cry-V96 (TUBB2A, TUBB). These have been extensively studied in visual and neuronal phenotypes, including AD [65–69]. Polymeric cytoskeletal fibers were among the top cellular components identified by GO annotation (Supplementary Table S1). Thus, cry-V96 shares the same molecular target as normal tau protein, which is thought to stabilize microtubules under stress [97]. Also relevant, enrichment of UBE2H [73] and CPS1 [74, 75] by cry-V96 suggests engagement of ubiquitin-mediated proteostasis and nitrogen metabolism oxidative stress pathways, respectively. Both are implicated in neurodegenerative disorders, suggesting the potential for cry-V96 in counteracting protein dysregulation during cellular stress. Additionally, enrichment of PTGES3, a Hsp90 co-chaperone, may indicate involvement of cry-V96 in protein folding machinery [76]. Lastly, the extracellular protein A2M has been linked to reducing β-amyloid toxicity and limiting fibril formation [78, 98], while EIF1AD knockdown increases tau biosensor cell activity [60]. Thus, this proteomics assessment of cry-V96 enrichment identified multiple putative interactions with relevance to AD.

Our subcellular localization studies revealed that cry-ELPs exhibit nuclear localization under basal conditions, which increased significantly following stress. In contrast, control ELPs showed no cellular uptake in either basal or stressed conditions. These findings indicate that the mini-αB-crystallin domain promotes cellular association and intracellular trafficking, rather than stress alone driving non-specific uptake. The mini-αB-crystallin peptide (residues 73–92) contains the core client-binding region, which retains chaperone activity independent of N-terminal phosphorylation sites. Phosphorylation of those sites have been linked to intracellular redistribution [99, 100]. Moreover, αB-crystallin lacks a classical nuclear localization signal [101], yet can accumulate in the nucleus during stress through interactions with misfolded proteins [102]. Our team recently [22] reported that cry-ELPs protect cells from lysosomal membrane disruption. Under H_2_O_2_-induced stress, both cry-V96 and cry-SI had reduced lysosomal colocalization. These findings support a model in which cry-ELPs coordinate intracellular trafficking to reach targets relevant to ER stress. GO analysis of proteins enriched by cry-V96 also identified significant targets in the nucleus and ER which may play roles in intracellular trafficking (Supplementary Table S1). One notable nuclear protein enriched by cry-V96 is SUB1, which protects genomic DNA from oxidative damage [79]. This observation (Fig. 5A, B) is intriguing since cry-V96 translocates to the nucleus during ER stress. As such, nuclear localization may facilitate interactions with stress-responsive transcriptional or DNA-repair pathways.

Thermoresponsive biomaterials such as ELPs are a promising strategy to prolong therapeutic residence within the eye. In this study, cry-V96 demonstrated biological activity across multiple mechanistic assays. Chaperone activity was confirmed at 4 °C and room temperature, where ELPs remain soluble, while cell-based assays were performed at 37 °C, where ELPs phase-separate. These findings demonstrate that cry-ELPs retain biological activity in both the soluble and coacervated states relevant to physiological temperature. Coacervation of ELPs is intended to enhance intraocular retention, provide sustained therapeutic exposure, and address key limitations with rapid clearance of intravitreal peptide therapeutics. Widespread clinical use of intravitreal administration allows direct access to the posterior segment of the eye and is routinely used for the treatment of retinal diseases, including AMD [103]. In this study, the mini-cry peptide was incorporated into two thermosensitive ELP architectures previously characterized for their physicochemical properties and biological activity [22]. Prior pharmacokinetic studies [21] demonstrated intravitreal cry-SI exhibited 14 days of PK/PD activity compared to free mini-cry peptide, which became undetectable after one day. Though cry-SI and cry-V96 share a similar molecular weight and *T_t_*, cry-SI is composed from a diblock architecture. Without mini-cry, the SI diblock assembles micelles between room temperature and ~ 70 °C [22, 104]. In this regime, the hydrophobic isoleucine block phase-separates, while the hydrophilic serine block retains solubility. In contrast, mini-cry causes cry-SI to undergo bulk phase separation upon collapse of its hydrophobic block; however, it remains possible that the hydrophilic block remains solvated within coacervate. While more work is needed to clarify this, diblock architecture correlates with worsened stability and reversibility of cry-SI assembly in comparison to cry-V96 (Fig. 1). Thus, cry-V96 was selected for further evaluation here because its monoblock architecture is structurally simpler and less susceptible to aggregation than cry-SI [105]. To better approximate its potential for human ocular pharmacokinetics, the present study evaluated cry-V96 pharmacokinetics in NZP rabbits. cry-V96 formed visible coacervate depots following intravitreal injection that persisted for at least one month (Fig. 7A).

Although these findings support the therapeutic potential of cry-V96, this study has limitations. First, this study is limited by lack of evaluation in a disease-relevant AMD model. Rabbit pharmacokinetic studies demonstrate prolonged intraocular retention; however, mechanism was only assessed in vitro using human models of the RPE. Our team previously confirmed in vivo efficacy of cry-SI using the sodium iodate murine model of RPE disruption [24]. Future preclinical studies should therefore assess efficacy, dose dependence, repeated-dose tolerability, retinal function, inflammatory responses, histopathologic changes, and potential long-term off-target interactions. A second limitation is that depot placement obscured part of the retina based on IR reflectance (Fig. 7A). We chose to administer depots within the visual field, because this allows non-invasive observation during its one-month dissolution. Future efficacy studies should evaluate depots placed outside the intra-ocular optical path or in another compartment, such as the sub-conjunctival space. Despite this, animals maintained normal vital signs and a lack of change to the retinal thickness, which can be sensitive to inflammation. Intravitreal administration is widely used in clinical procedures that require direct access to the posterior segment of the eye and is routinely used for retinal diseases, including AMD [103]. These findings are consistent with the established clinical feasibility demonstrated by long-acting anti-VEGF therapies [106]. A third limitation is that we do not know the mechanism of action by which exogenous cry-ELPs enter cells and modulate protection against oxidative and ER stressors. That limitation motivated the proteomics study herein (Table 1). Although proteomic analysis identified several candidate interacting proteins and pathways, these observations remain correlative and require validation using complementary biochemical and functional approaches. Fourth, it is unknown how sustained non-specific chaperone activity in the eye may affect ocular homeostasis. Although sustained activity of molecular chaperones may produce unintended effects, findings obtained with full-length chaperones should not be directly extrapolated to the peptide in the present study. mini-cry is a 20-amino-acid chaperone peptide extracted from αB-crystallin that lacks the higher-order structure of the full-length protein. This sequence has not been identified as a VEGF-binding site, and there is currently no evidence that it directly binds or stabilizes VEGF [107]. Nevertheless, αB-crystallin-derived peptides have been reported to interact with other regulatory proteins, including fibroblast growth factor-2 (FGF-2), indicating that interactions with growth factors cannot be excluded [107, 108]. Similarly, the context-dependent effects of chaperone (Hsp70) overexpression reported by Jiang and coworkers [109] involved a full-length, ATP-dependent chaperone and are not directly comparable to exogenous mini-cry formulations. Nevertheless, multi-dose studies should assess retinal structure and function, inflammatory and vascular responses, FGF-related effects, and potential off-target interactions to establish long-term ocular safety. Further development of intravitreal ELP therapeutics, including crystallin-based depots, will need to focus on quantifying and overcoming these limitations.

## Conclusion

This manuscript reports that cry-ELP fusions combine the beneficial delivery properties of ELPs with the chaperone activity of αB-crystallin. cry-V96 emerges as the lead construct, which exhibits consistent in vitro biological activity. Exogenous cry-V96 shows enhanced cellular uptake with stress-dependent nuclear localization, which may attenuate ER stress. Importantly, cry-V96 also forms a stable, long-lasting intravitreal depot, without altering the retina thickness. Future studies are needed to examine its mechanism of cellular uptake, endolysosomal escape, and nuclear translocation, particularly because the mini-αB-crystallin peptide lacks a defined nuclear localization sequence. While proteomic analysis identified numerous interacting proteins and pathways, those findings are correlative and require validation using complementary biochemical and functional studies. Lastly, rabbit pharmacokinetic studies demonstrated prolonged intraocular retention; however, efficacy was not evaluated in a disease-relevant AMD model. These findings position cry-V96 as a promising strategy for targeting protein aggregation and stress pathways in retinal diseases, while highlighting ELPs as an emerging platform for sustained drug delivery.

## Supplementary Material

Supplemental A

Supplemental B

Supplemental Proteomics Hits

The online version contains supplementary material available at https://doi.org/10.1007/s13346-026-02210-w .

## Figures and Tables

**Fig. 1:**
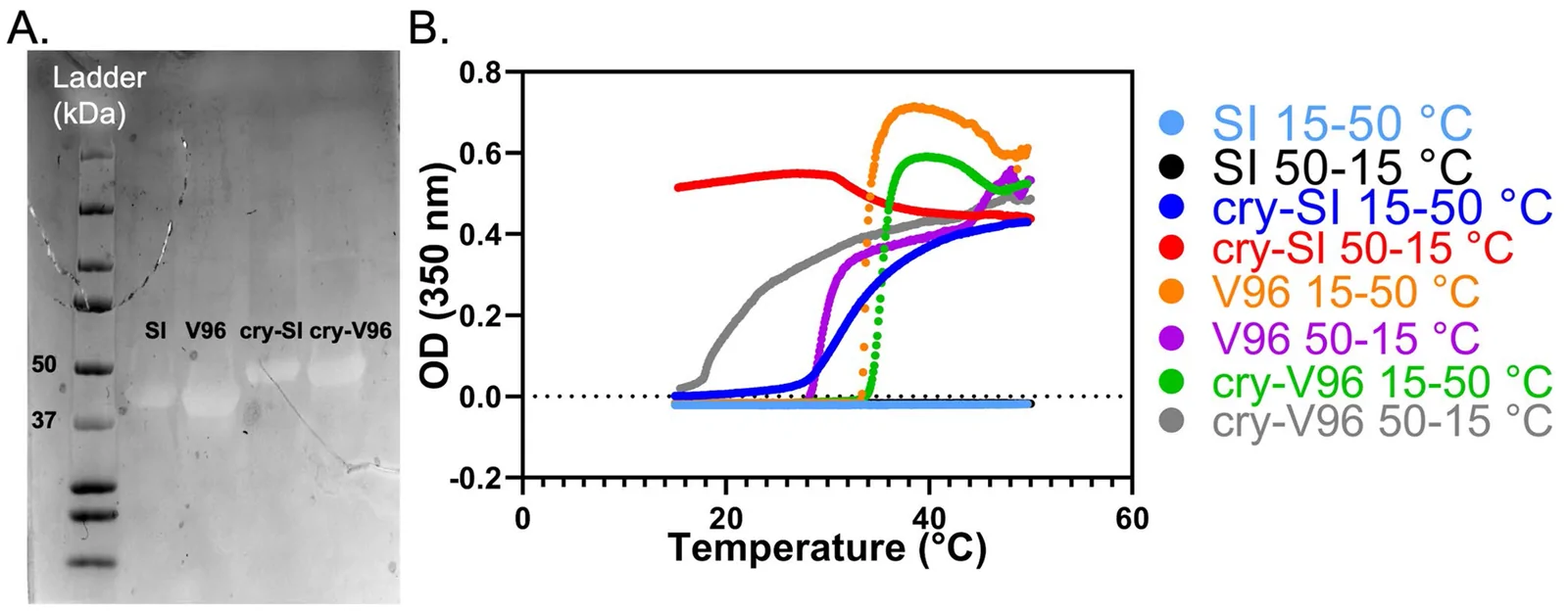
cry-V96 displays thermoresponsive reversibility. **A** SDS-PAGE with Copper(II) chloride staining (white) confirmed the purity of bacterially expressed ELPs, with the major bands near the expected molecular weights (39.6, 39.6, 42.1, and 42 kDa) for SI, V96, cry-SI and cry-V96 respectively. A prestained ladder was selected (black). **B** Thermal responses were measured by UV-Vis spectrophotometry during heating (15–50 °C) and cooling back to 15 °C. cry-SI, V96, and cry-V96 showed OD increases, with *T*_*t*_ values of 31, 34, and 35 °C, respectively. V96 and cry-V96 returned to baseline; however, during cooling cry-SI remained opaque, demonstrating a more extensive hysteresis

**Fig. 2:**
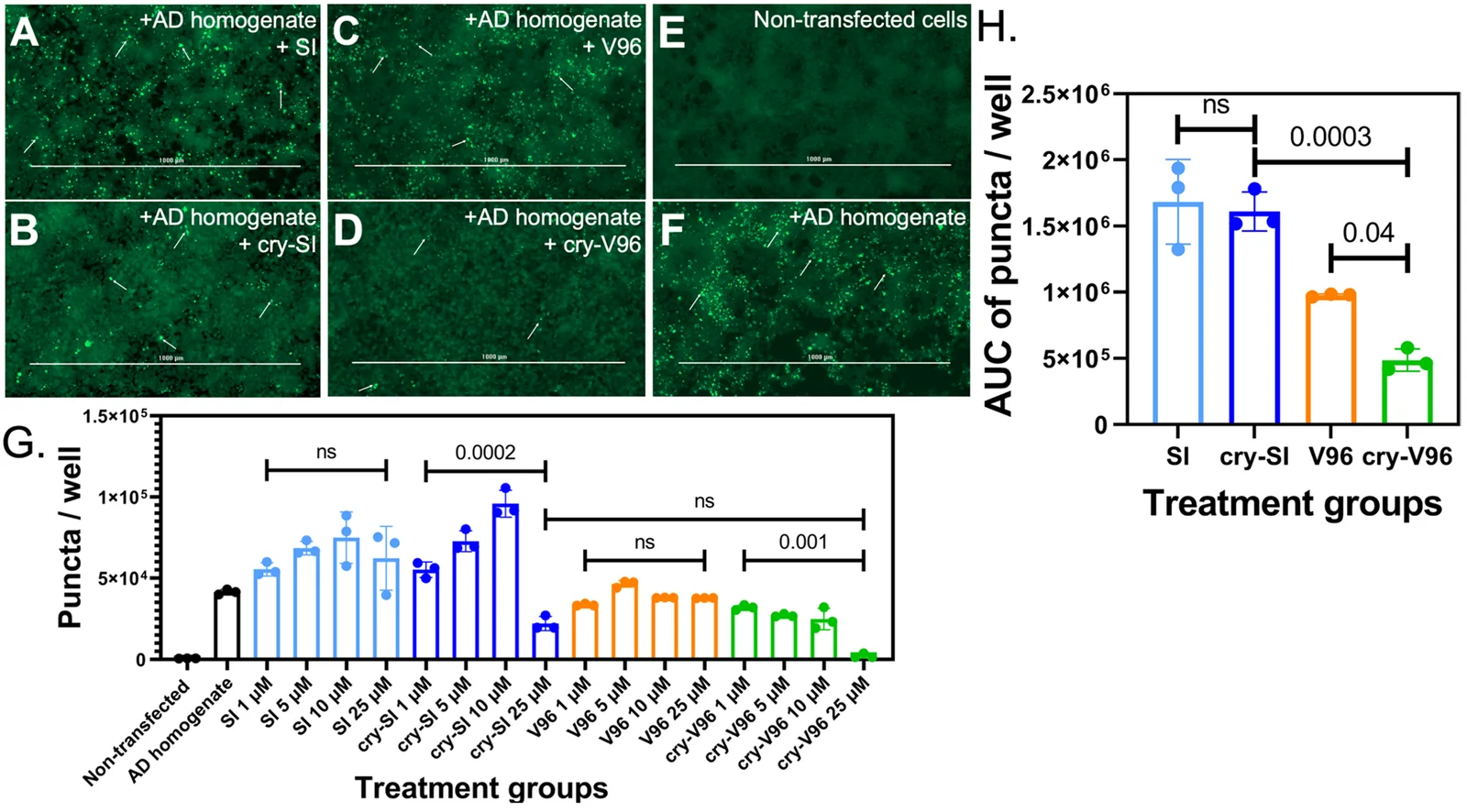
Exogenous cry-V96 inhibits intracellular tau seeding in HEK293T biosensor cells. Cells expressing a YFP-fused K18 tau fragment containing the P301S and V337M aggregation-prone mutations were transduced with AD crude brain homogenate preincubated for 24 h with 25 μM (**A**) SI, (**B**) cry-SI, (**C**) V96, or (**D**) cry-V96 and imaged at low magnification (scale bar = 1000 μm). **E** Non-transduced cells and (**F**) treated with AD homogenate alone were the negative and positive controls, respectively. Tau-positive cells have intense green puncta (white arrows), while negative cells show low-level fluorescence. **G** Control ELPs (SI, V96) did not reduce puncta. In fact, puncta increased with SI and cry-SI concentrations up to 10 μM. This was not observed for V96. Only at the highest concentration, did cry-SI reduce puncta. In contrast, cry-V96 demonstrated consistent dose-dependent reduction of puncta. Few puncta remained at 25 μM cry-V96. **H** AUCs were calculated across the concentration gradient, which revealed that only cry-V96 inhibited tau aggregation. (One-way ANOVA with Tukey’s post hoc test; *p* values shown, mean ± SD, *n* = 3)

**Fig. 3:**
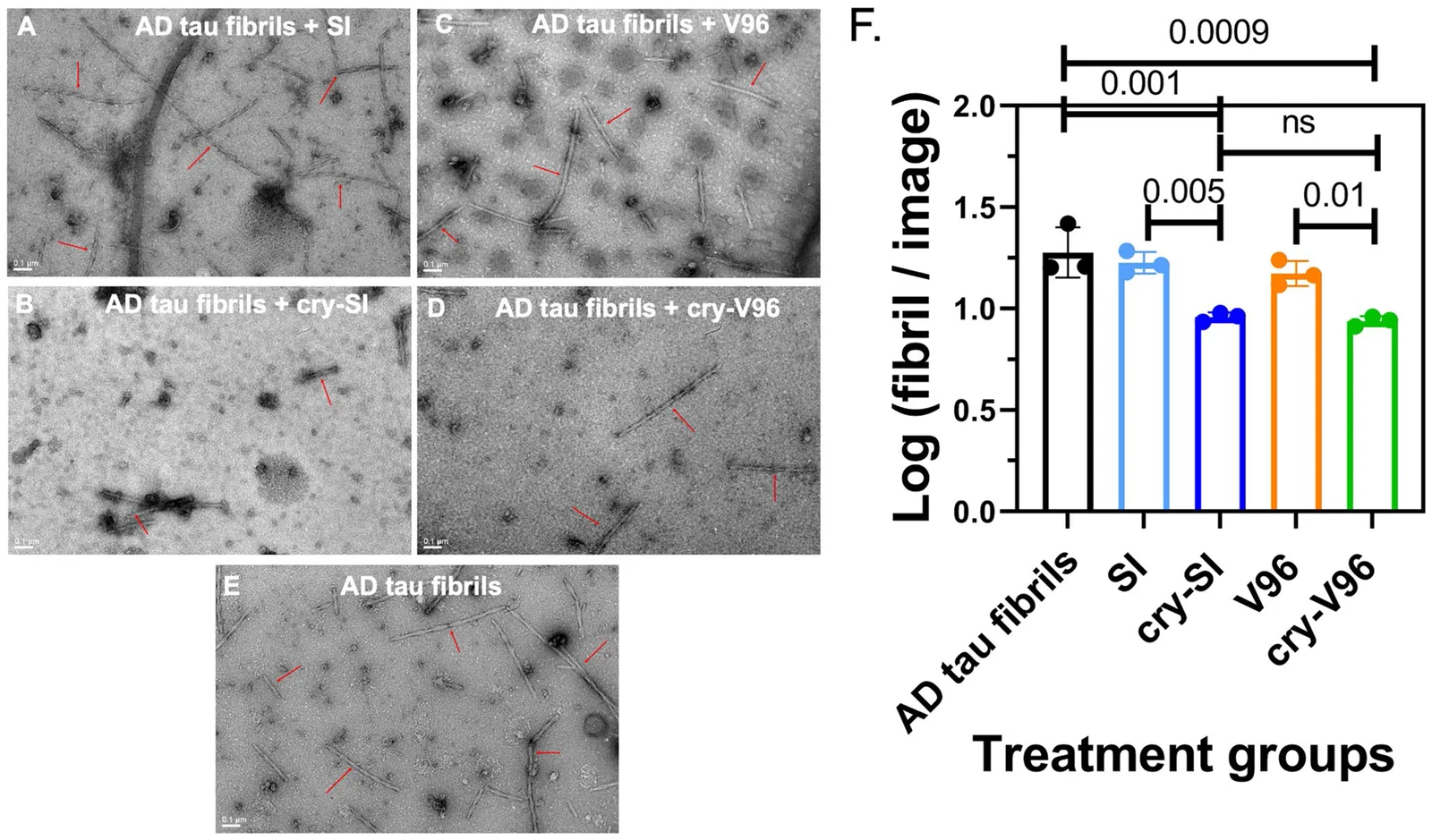
cry-ELP fusions disaggregate AD tau fibrils. TEM was used to quantify tau fibrils treated with (**A**) SI, (**B**) cry-SI, (**C**) V96, and (**D**) cry-V96. Fibrils were quantified from three sets of images (*n* = 50 images per set, scale bar = 0.1 μm), with representative fibrils indicated by red arrows. **E** Non-treated tau fibril was used as control. **F** Only cry-ELPs significantly reduced fibril counts, compared to control ELPs and untreated samples (One-way ANOVA with Tukey’s post hoc test; *p* values shown, mean ± SD, *n* = 3), demonstrating their ability to disaggregate tau fibrils and inhibit further assembly

**Fig. 4:**
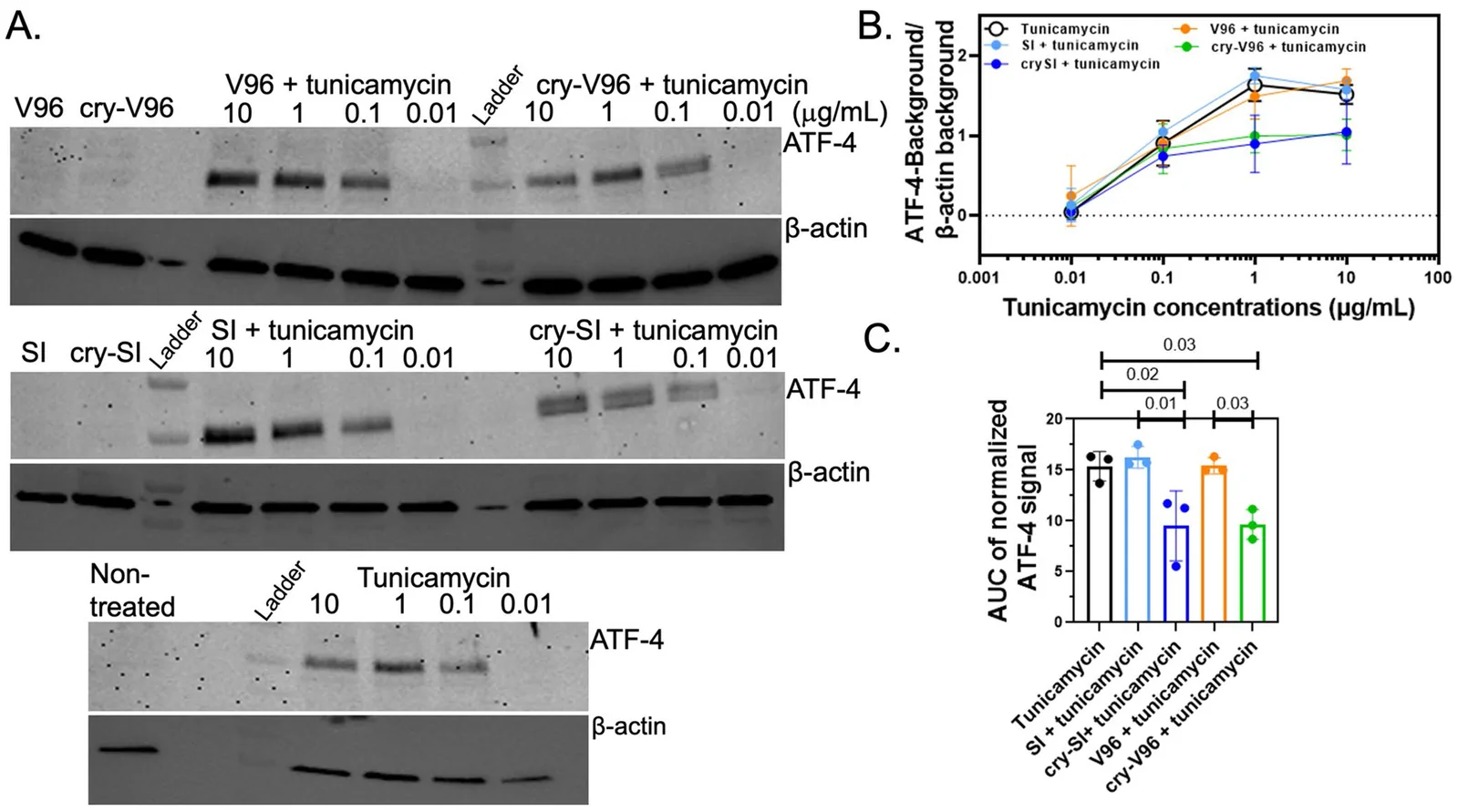
cry-ELPs suppress ATF-4 activation in ARPE-19 cells under tunicamycin challenge. **A** Western blotting was performed to assess ATF-4 regulation in starved ARPE-19 cells. Cells were either untreated or exposed to a range of tunicamycin concentrations for 24 h, following a 24 h pretreatment with SI, V96, cry-SI or cry-V96 and assessed by Western blot. **B** Background-subtracted integrated intensities of ATF-4 bands were normalized to β-actin and plotted as a function of tunicamycin concentration (Eq. 1). ATF-4 signal was reduced by cry-ELPs above tunicamycin concentrations of 0.01 μg/mL. **C** AUC of the normalized signal vs. tunicamycin concentration was calculated for each replicate and compared by one-way ANOVA with Tukey’s post hoc test (*p* values shown, mean ± SD, *n* = 3)

**Fig. 5:**
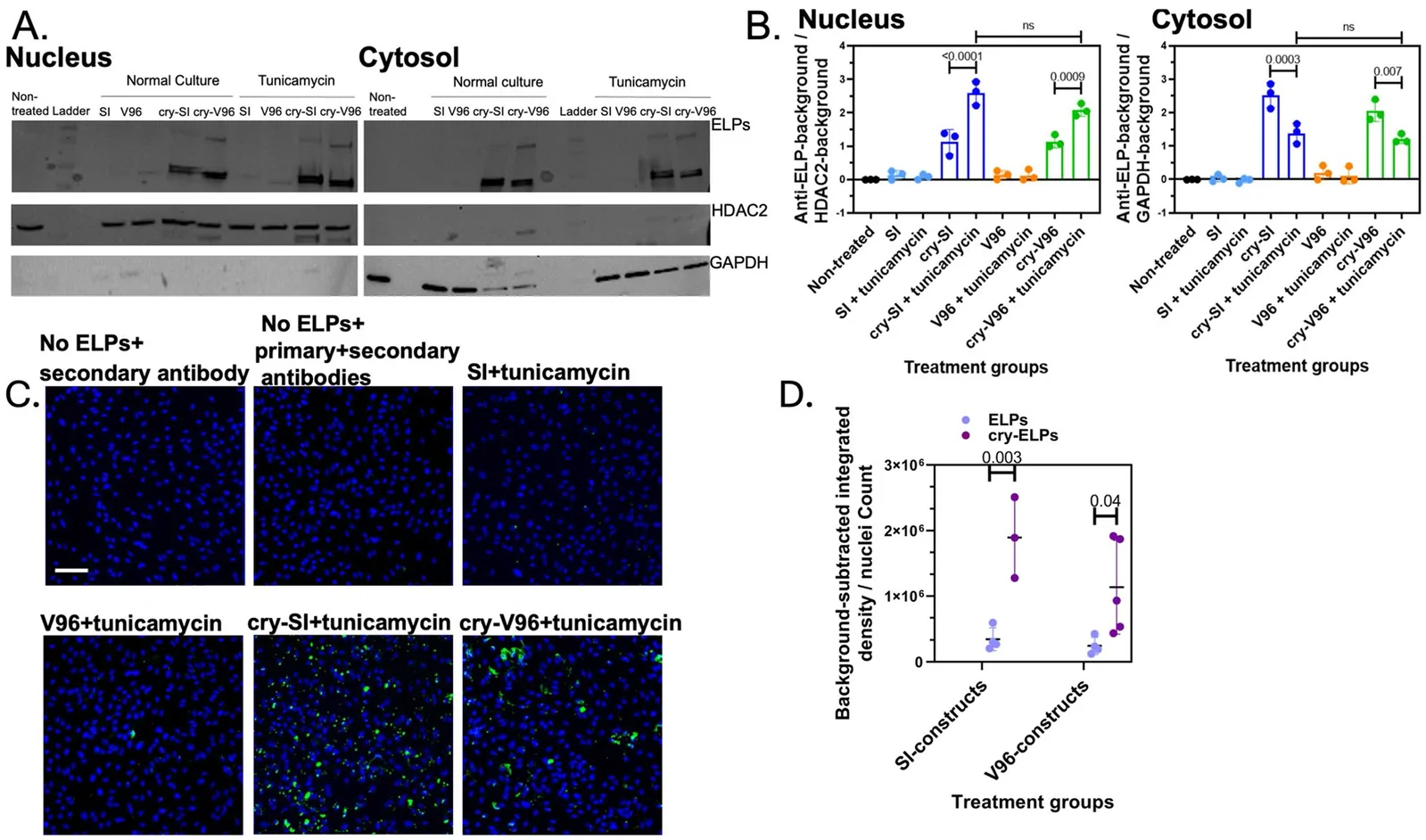
cry-ELPs show enhanced cellular association and nuclear localization under tunicamycin stress. **A** ARPE-19 cells were serum-starved, incubated with 25 μM ELPs for 24 h, challenged by 0.1 μg/mL tunica-mycin for 24 h, fractionated, and analyzed by Western blot using anti-ELP antibodies. Fraction purity was confirmed using HDAC2 (nuclear marker) and GAPDH (cytosolic marker). As expected, HDAC2 was expressed in the nucleus (left), while GAPDH appears in cytosol extracts (right). The minimal GAPDH in nuclear extracts is consistent with literature findings of nuclear proteins [43]. **B** Nuclear/cytosolic signals were normalized to these markers, respectively (Eq. 1). Cry-ELPs showed increased nuclear and cytosolic association compared to control ELPs (SI and V96), with tunicamycin increasing nuclear enrichment and decreasing cytosolic levels (One-way ANOVA with Tukey’s post hoc test, *p* values shown, mean ± SD, *n* = 3). **C** Confocal microscopy confirmed cellular association of cry-ELPs following 24 h tunicamycin treatment. Images were acquired at 20· (scale bar = 50 μm, Nuclei: blue, ELP: green). **D** Quantification of integrated fluorescence density normalized to the nuclei count confirmed significantly higher cellular association of cry-ELPs vs. control ELPs (Two-way ANOVA with Sidak’s post hoc test, *p* values shown, mean ± SD, *n* = 3–5)

**Fig. 6:**
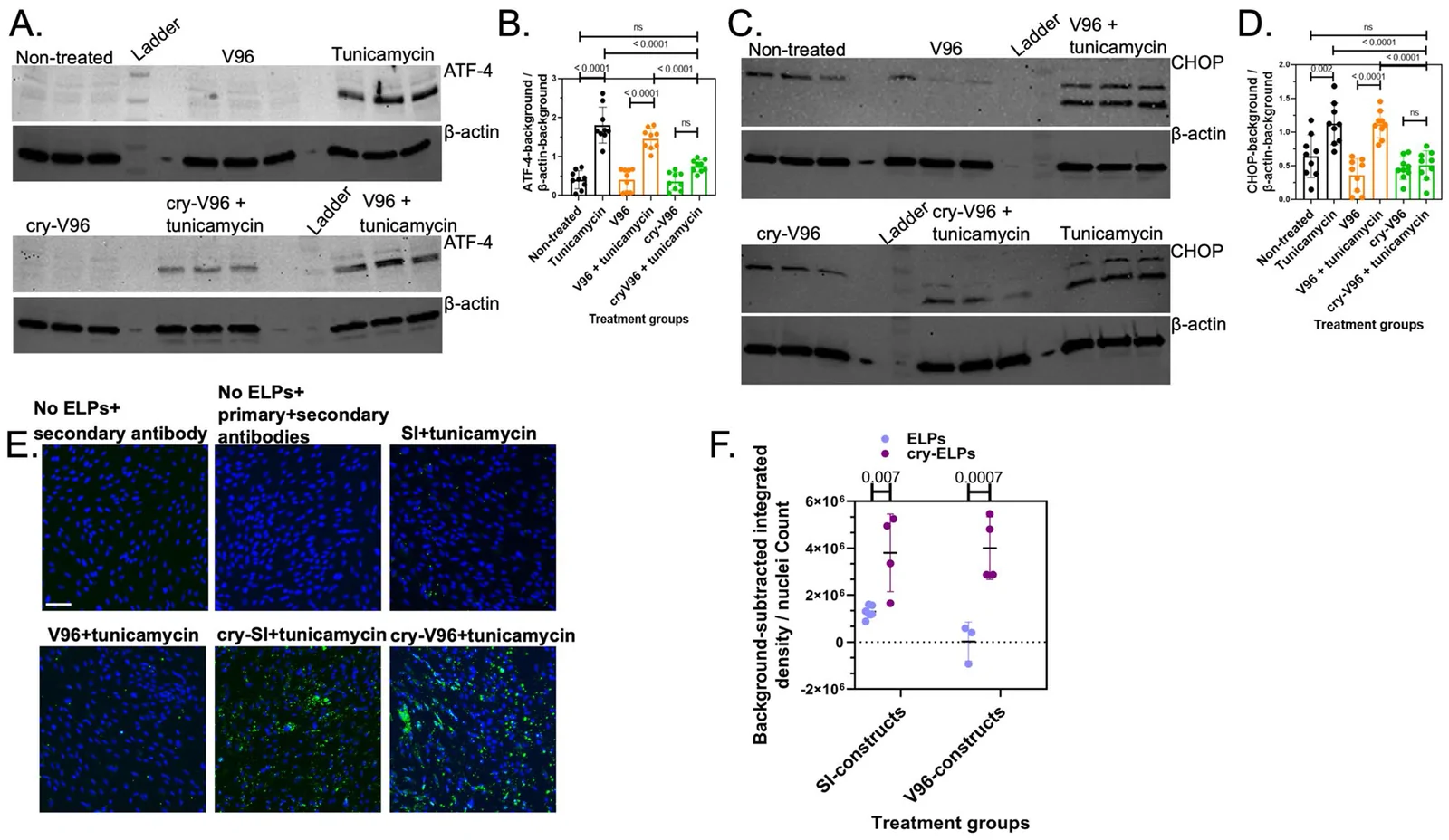
cry-V96 suppresses ATF-4 and CHOP signaling while enhancing cellular association on tunicamycin-challenged hRPE cells. hRPE monolayers were serum-starved overnight, exposed to 25 μM of V96 or cry-V96, challenged with tunicamycin. Lysates were probed with (**A**, **B**) ATF-4 and (**C**, **D**) CHOP antibodies. Band intensities were normalized to β-actin (Eq. 1). **B** ATF-4 and (**D**) CHOP upregulation induced by tunicamycin were significantly decreased by cry-V96, in comparison to V96 control (One-way ANOVA with Tukey’s post hoc test, *p* values shown, mean ± SD, *n* = 9 independent replicates). **E** Cellular uptake of cry-ELPs in tunicamycin-challenged hRPE was assessed by confocal microscopy (20×, scale bar = 50 μm) using anti-ELP primary (green) with nuclei counterstained with Hoechst (blue). Untreated cells (no ELPs) incubated without antibodies were imaged to determine thresholds that distinguish signal from background. **F** Quantification of fluorescence normalized to nuclei number confirmed significantly higher cellular association of cry-ELPs compared to control ELPs (Two-way ANOVA with Sidak’s post hoc test, *p* values shown, mean ± SD, *n* = 3–6)

**Fig. 7:**
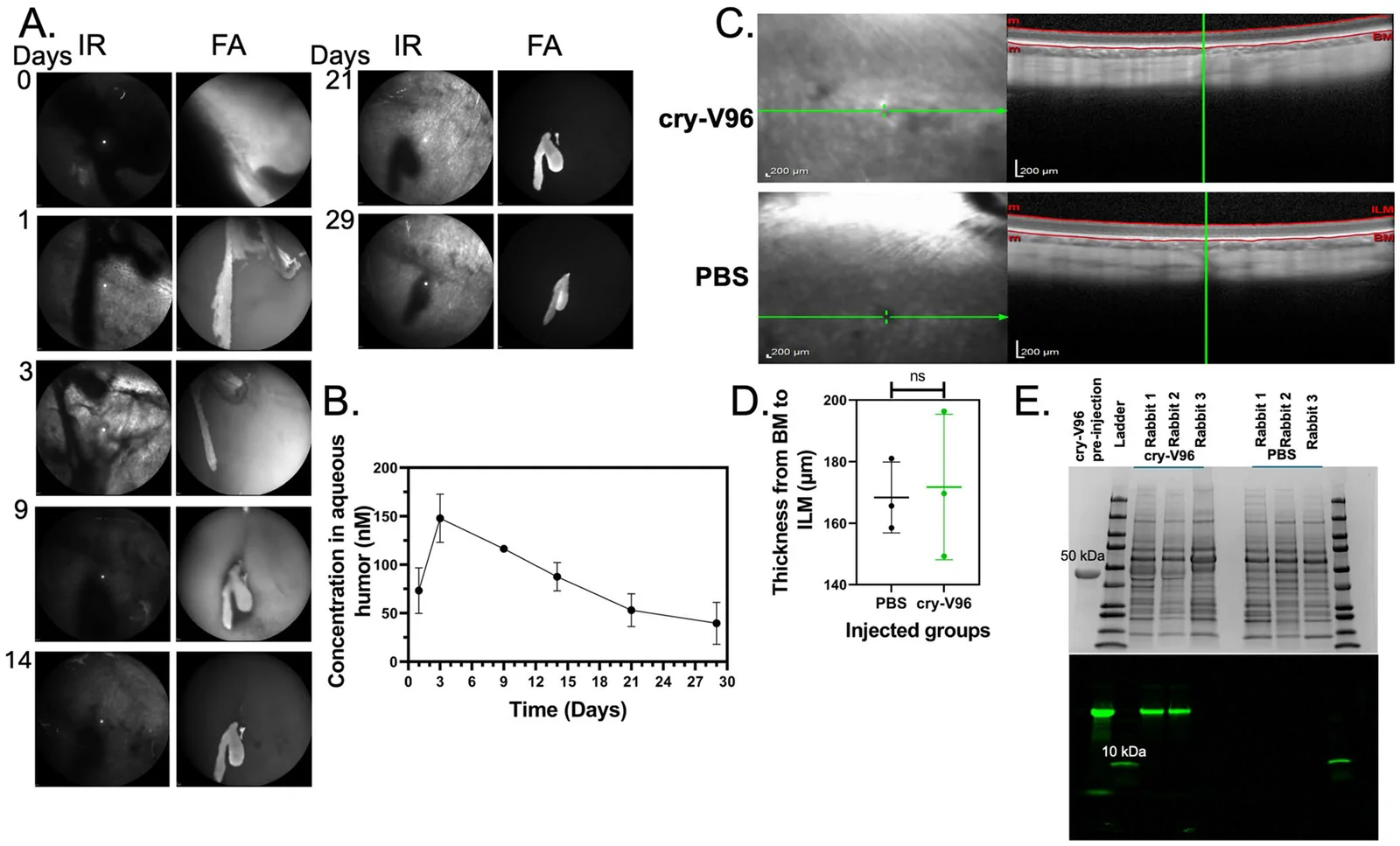
Intravitreal cry-V96 persists in vitreous humor for up to one month. **A** Spectralis OCT was used to evaluate the pharmacokinetics of fluorescent cry-V96 in rabbits following intravitreal injection. FA imaging of a representative depot (rabbit 1) over a 29-day study revealed a diffuse signal (whitish background) from days 1–14 (scale bar = 200 μm). Imaging data for all three rabbits are in Supplemental Fig. S4. **B** Aqueous humor concentrations of the tracer were detectable for the entire study; however, peak levels were detected day 1 after administration. **C** Retinal cross-sections were obtained on day 29 prior to euthanasia (top left). Retinal thickness was measured from Bruch’s membrane (BM) to the internal limiting membrane (ILM) (top right) and quantified (scale bar = 200 μm). **D** No significant difference in retinal thickness was observed between cry-V96 and PBS injected eyes (Student’s *t*-test, *p* values shown, mean ± SD, *n* = 3 per eye). **E** Vitreous humor samples were analyzed by SDS-PAGE using Coomassie staining (upper panel) and fluorescence (bottom panel), in comparison with preinjection fluorescent cry-V96 and PBS-injected eyes

**Table 1:** Subset of proteins enriched in cry-V96-treated hRPE cells under tunicamycin-induced ER stress

Enriched proteins	Abundance fold (cry-V96/V96)	Adjusted *p* values	Chaperone-validated interaction	Mechanistic function
CHMP4B	48	2 · 10^−7^	Hsp90β [49], *K_d_ = 168 nM	ESCRT transport, multi-vesicular body assembly
CHMP4A	20	1 · 10^−3^	N/A	[50], ER remodeling [51]
CHMP5	17	3 · 10^−3^		
SMAP	28	4 · 10^−5^		Clathrin vesicle trafficking [52], endosomal vesicle recycling [53]
MYL9	26	1 · 10^−4^		Cytoskeletal dynamics
MYL12B	18	2 · 10^−3^		[54, 55]
EIF1AD	100	2 · 10^−16^		Translation regulation and cell survival [56–60]
EIF1AX	100	2 · 10^−16^		
EIF1AY	85	2 · 10^−10^		
SRSF1	25	1 · 10^−4^		Gene expression [61, 62]
SRSF3	21	6 · 10^−4^		
RCN1	21	6 · 10^−4^		Calcium homeostasis [63]
ANP32B	16	5 · 10^−3^		Cytoprotection modulator [64]
TUBB2A	13	2 · 10^−2^		Microtubule assembly
TUBB	11	5 · 10^−2^	HspB5/αB-crystallin [70], Hsp90 [71, 72]	[65–69]
UBE2H	13	2 · 10^−2^	*K_d_ = 138 nM	Protein degradation [73]
CPS1	48	2 · 10^−7^	N/A	Nitrogen detoxification [74, 75]
PTGES3	17	3 · 10^−3^	Hsp90 [76]	Proteostasis [76]
A2M	11	1 · 10^−2^	HspA5/GRP78 [77]	Amyloid scavenger [78]
SUB1	15	9 · 10^−3^	*K_d_ = 9 nM	Transcription regulation [79]

* Equilibrium binding experimentally quantified using fluorescence anisotropy (Supplemental Fig. S5)

## Data Availability

A supplementary document includes additional information related to the cell-line viability, ocular pharmacokinetics, and gene-ontology of proteomic interactions. In addition, a spreadsheet file been attached that includes information about the most significant proteins identified in the proteomics study with cry-V96. Additional data used to prepare this study are available through contact with the corresponding authors.
