# Supplementary material for: αB-crystallin elastin-like polypeptides for sustained ocular drug delivery
Source: Drug Deliv Transl Res. Author manuscript; Available in PMC 2026 Oct 6. (PMC13641684; doi:10.1007/s13346-026-02210-w)
Supplement: Supplementary file 1 [file NIHMS2211083-supplement-Supplemental_A.pdf]

Supplemental Appendix A

αB-crystallin elastin-like polypeptides for sustained ocular drug delivery

Sara Aly Attia[1], Amanda M. Tse[1], Ashley D. Ramirez[1], Maryam Izadiar[1], Ying Liu[2], Hao Guo[1], Angela Albanese[1], Whitaker Cohn[3], Adrienne W. MacKay[4], Paul M. Seidler[1], Ram Kannan[5,6], Parameswaran G. Sreekumar[5*], John Andrew MacKay[1,2,7 *]

[1] Department of Pharmacology and Pharmaceutical Sciences, University of Southern California Mann School of Pharmacy and Pharmaceutical Sciences, Los Angeles, CA 90033, USA
[2] Department of Ophthalmology, Roski Eye Institute, University of Southern California Keck School of Medicine, Los Angeles, CA 90033, USA
[3] Titus Family Department of Clinical Pharmacy, University of Southern California Mann School of Pharmacy and Pharmaceutical Sciences, Los Angeles, CA 90033, USA
[4] Department of Population and Public Health Sciences, University of Southern California Keck School of Medicine, Los Angeles, CA 90033, USA
[5] Doheny Eye Institute, Pasadena, CA 91103, USA
[6] Stein Eye Institute, University of California Geffen School of Medicine, Los Angeles, CA 90095, USA
[7] Alfred E. Mann Department of Biomedical Engineering, University of Southern California Viterbi School of Engineering, Los Angeles, CA 90089, USA

*co-corresponding authors
John Andrew MacKay, jamackay@usc.edu
Parameswaran G. Sreekumar, sparameswaran@doheny.org

S1.

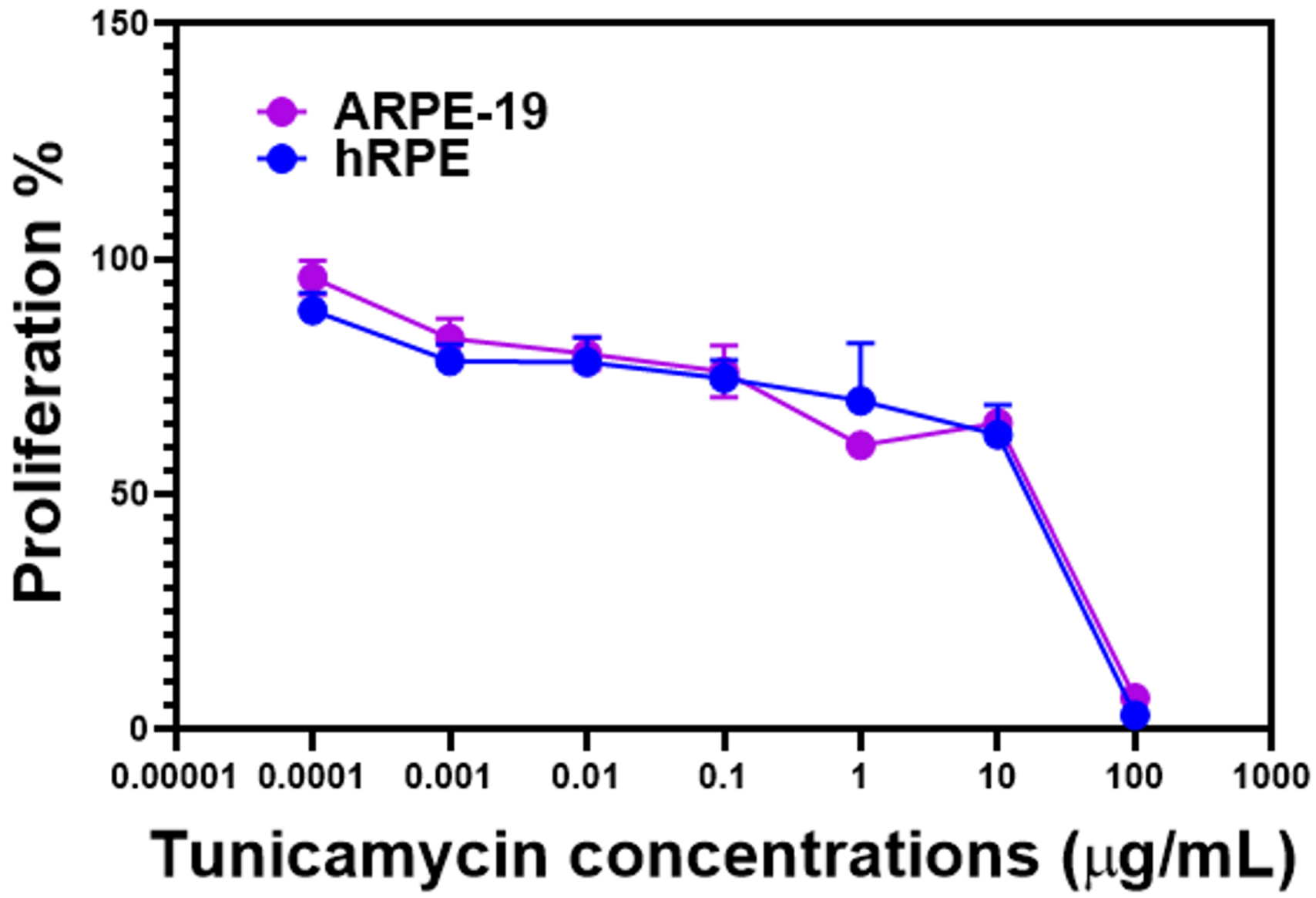

Supplementary Figure S1. Tunicamycin induces a dose-dependent reduction in metabolic activity in ARPE-19 and hRPE cells. ARPE-19 and hRPE cells were seeded independently in 96-well plates (Genesee Scientific #25-109) till grown to 60 % confluence. The next day, cells were serum-starved overnight in 1% FBS and then treated with tunicamycin (0.0001-100 μg/mL) in the same starved medium for 24 h. Following tunicamycin treatment, 10 μL of WST-1 (MilliporeSigma #5015944001) was added to each well and formazan absorbance was quantified at 450 nm using a H1 Hybrid Multi-Mode Reader. While concentrations (0.0001-100 μg/mL) showed a dose-dependent reduction in metabolic activity in ARPE-19 (96 ± 4, 83 ± 4, 80 ± 3, 76 ± 6, 60 ± 2, 65 ± 3, and 6 ± 3) and hRPE (89 ± 4, 78 ± 4, 78 ± 5, 75 ± 4, 69 ± 12, 62 ± 6, and 3 ± 2), respectively, only 100 μg/mL was lethal. These data justify using tunicamycin concentrations from 0.01-10 μg/mL to study sublethal cellular stress via modulation of the UPR through ATF-4 and CHOP signals (**Figs. 4, 6**). (Mean ± SD, n = 6)

S2.

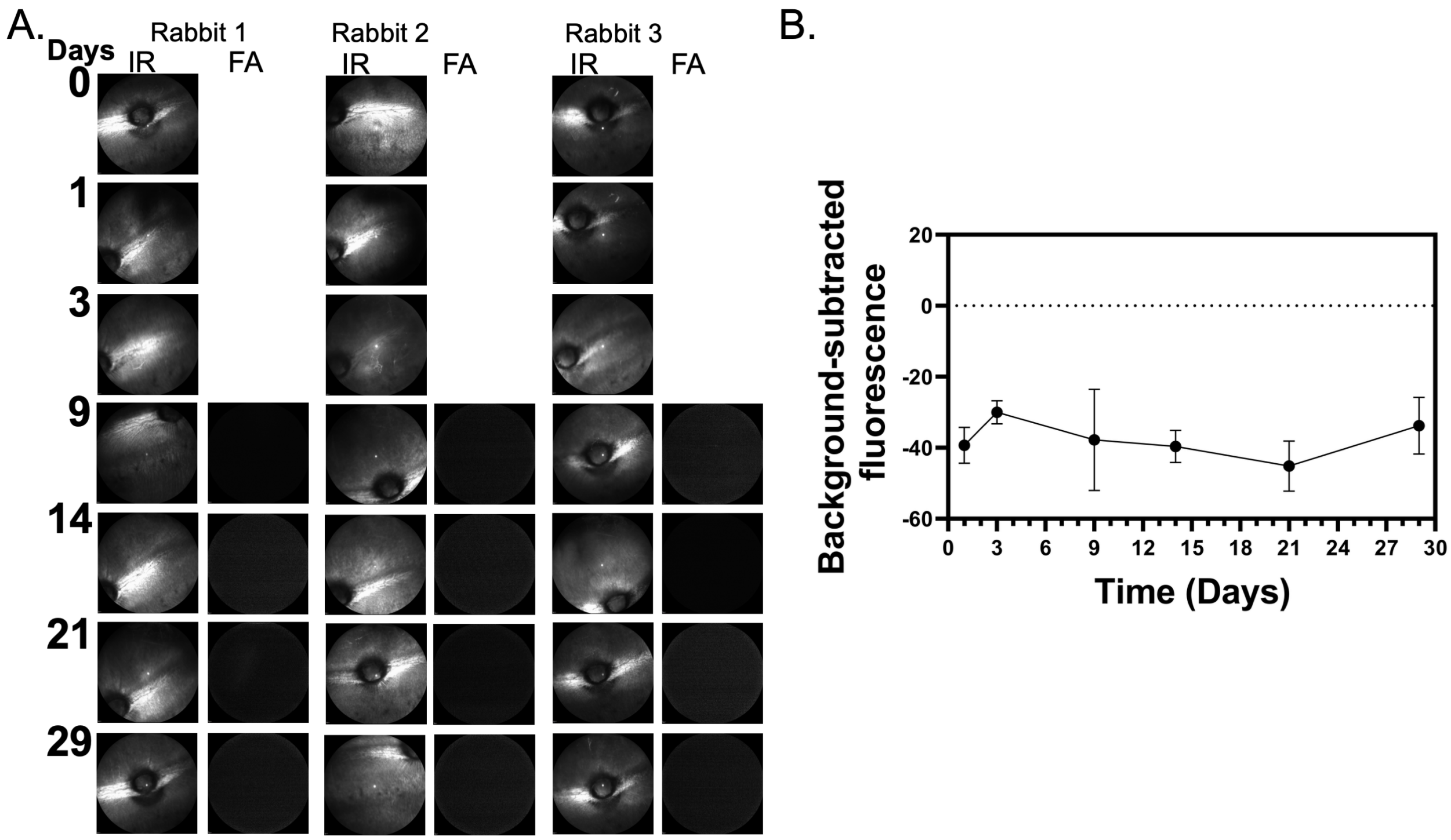

Supplementary Figure S2. OCT images of eyes (n = 3) injected with PBS and aqueous humor samples indicate no observable coacervates. A) Images acquired over 29 days demonstrated clear visualization of the optic nerve in the IR channel and a lack of signal (black) in the FA channel, which indicated the absence of green fluorescence. Only IR images were collected for the first 3 days. B) Background-subtracted raw fluorescence of the collected aqueous humor samples showed no change over time. Together, these results confirm that there was no cross-circulation of signal from the contralateral eye, which was injected with NHS-carboxyfluorescein-labeled cry-V96 (scale bar = 200 μm).

S3.

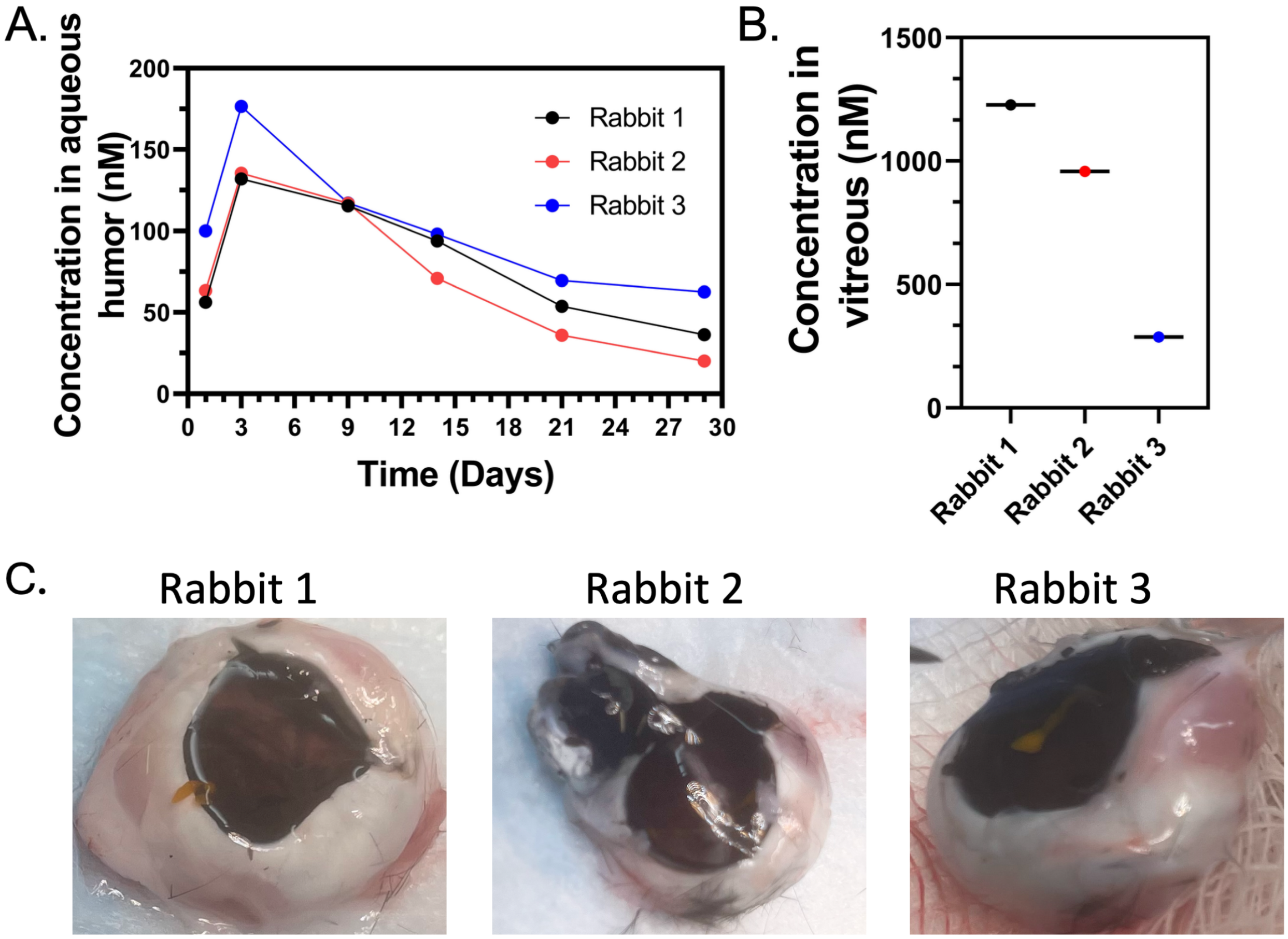

Supplementary Figure S3. cry-V96 demonstrates detectable levels in both aqueous humor and vitreous samples over 29 days. A) In the aqueous humor samples, rabbits 3, 1, and 2 showed the highest detectable fluorescent cry-V96 levels, respectively. B) Upon conclusion of the study, rabbit 3 had the lowest concentration detected in the vitreous sample (287 nM), which may correlate with increased absorption into the aqueous humor. In comparison, the vitreous concentrations for rabbits 1 and 2 remained relatively similar, at 1229 nM and 958 nM, respectively. Consistent with the plate reader results, rabbit 3 had 3-4 fold lower in vitreous concentration, relative to rabbits 1 and 2, respectively, which may align with the lack of band detection by SDS-PAGE (**Fig. 7E**). C) After removal of the cornea, the exposed vitreous enabled direct imaging of the remaining fluorescent depot (yellow).

S4.

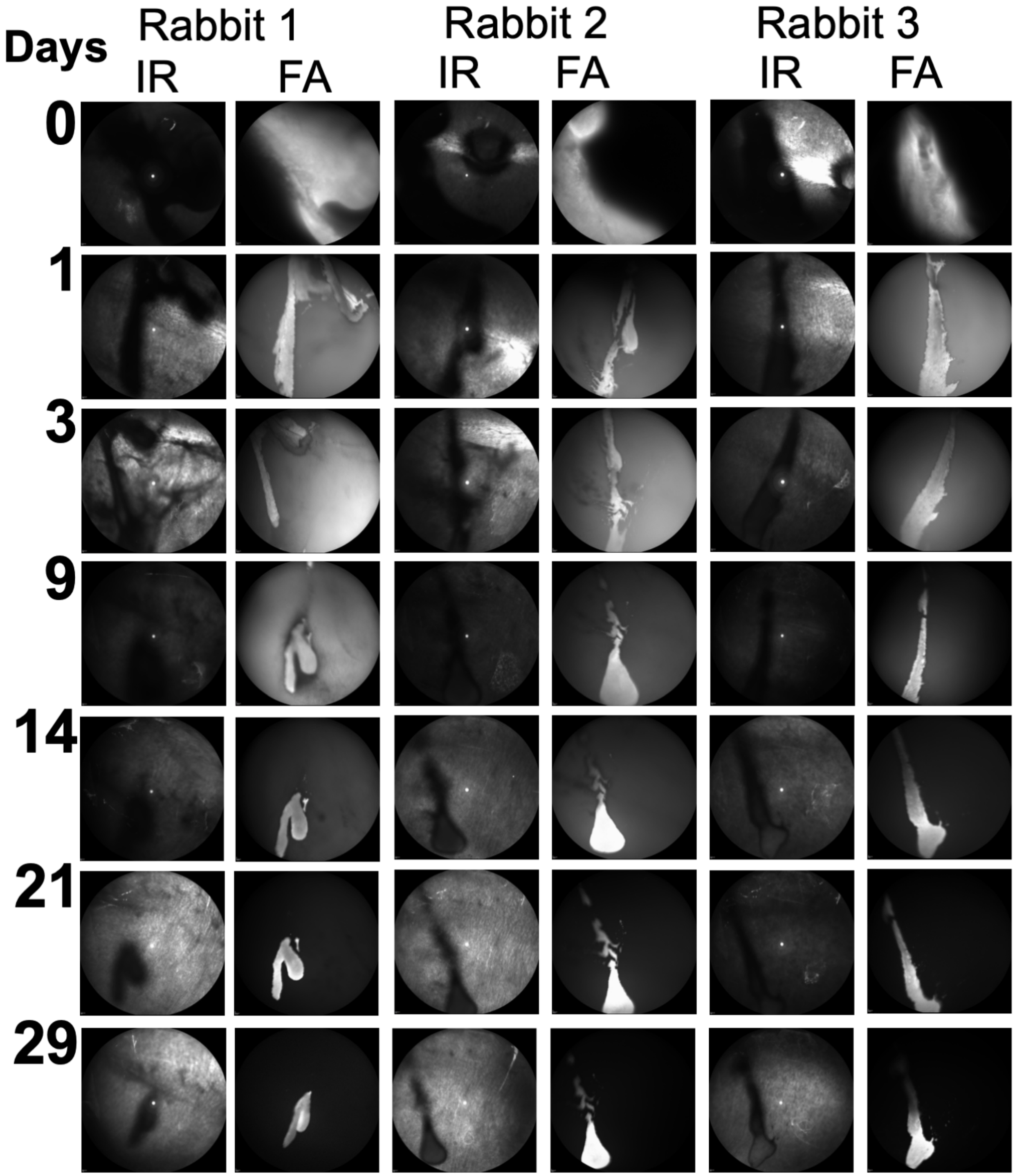

Supplementary Figure S4. Comparative view of Spectralis OCT IR and FA imaging over this study. When centered on the depot, all three rabbits exhibited relevant fluorescent cry-V96 depots up to day 29 (scale bar = 200 μm).

S5.

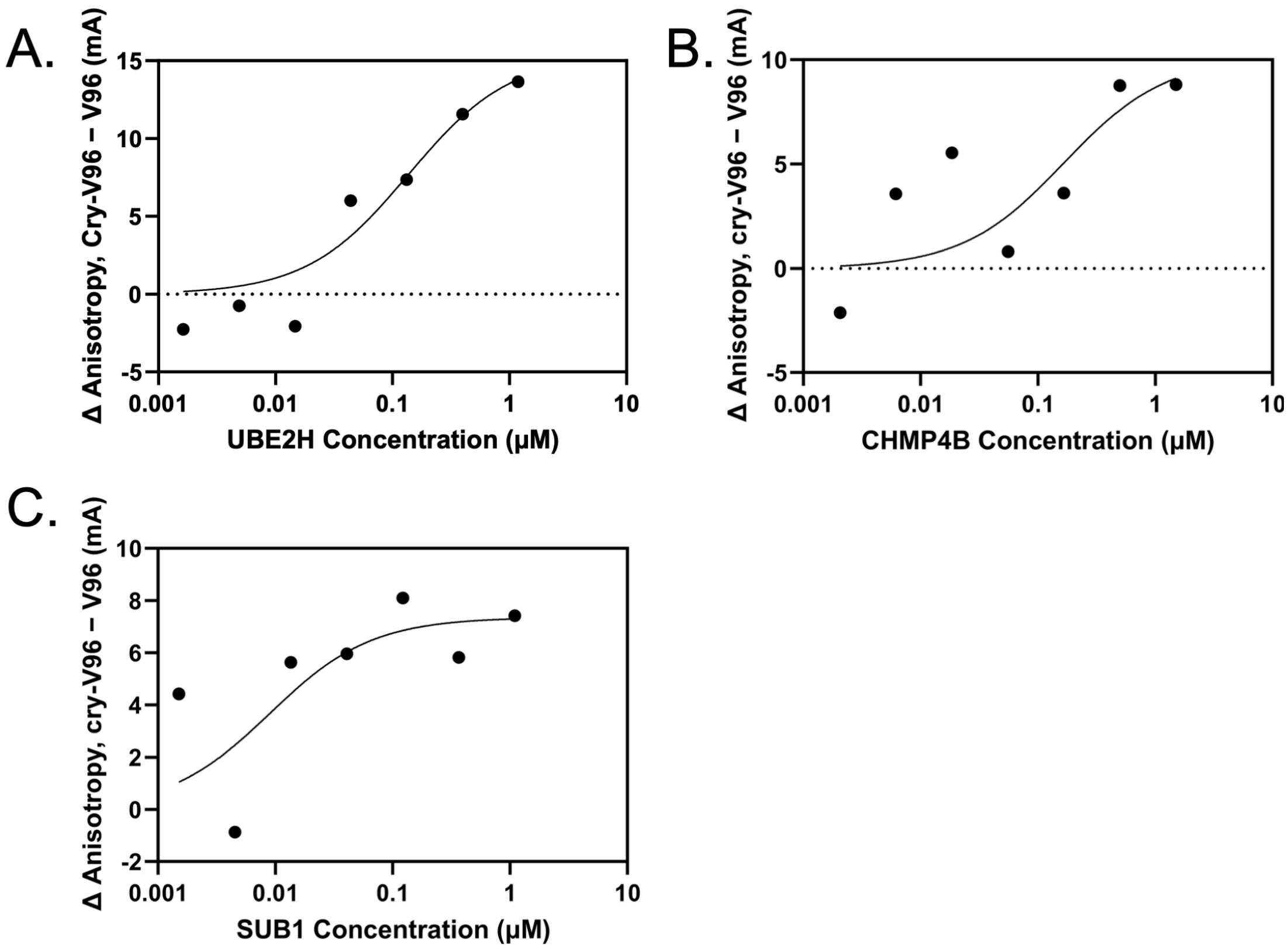

Supplementary Figure S5. Binding of UBE2H, CHMP4B, and SUB1 to cry-V96. Fluorescence anisotropy measurements (H1 Hybrid Multi-Mode Reader, equipped by fluorescence polarization filter cube) were performed in a 384-well black flat plates (Corning #3821) using 10 nM of fluorescein labelled-cry-V96 in the presence of increasing concentrations of A) UBE2H, B) CHMP4B, and C) SUB1, with 10 nM fluorescein labelled-V96 as the control. $r - r_0$ is the anisotropy at each concentration minus the anisotropy of the fluorophore alone. Data are presented as the difference in anisotropy, between cry-V96 and V96 [$\Delta(r - r_0)$, cry-V96 − V96]. The solid line represents the nonlinear regression fit to $r - r_0 = \Delta r_{\text{max}} C/(K_d + C)$, where C is the concentration of UBE2H, CHMP4B, or SUB1. The fitted Δr_{max} values were 15.4, 10.1, and 7.36 mA, with K_d values of 138, 168, and 9 nM, respectively.

Supplementary Table S1. GO identified significant pathways represented by proteins enriched by cry-V96 pulldown significantly greater than V96.

GO category*	Enriched pathway	*p* value	Enriched Proteins
Molecular Function	Myosin Heavy Chain Binding	4×10^{-4}	MYL9; MYL12B
	Myosin Binding	6×10^{-4}	MYL9; RAB6B; MYL12B
Biological Process	mRNA Cis Splicing, via Spliceosome	2×10^{-5}	SRSF1; SRSF3; CWC15
	Endosome to Lysosome Transport via Multivesicular Body Sorting Pathway	1×10^{-3}	CHMP4B; CHMP5
	Protein-Containing Complex Assembly	3×10^{-2}	YAP1; PTGES3; PDCL; CHMP4B; PDE4DIP
	Regulation of Programmed Cell Death	3×10^{-2}	ANP32A; ANP32E; ANP32B; CRYAB
Cellular Component	Nucleus	3×10^{-4}	YAP1; ANP32A; MAX; SRSF1; CTCF; PQBP1; EIF1AD; C1QBP; UBE2H; TUBB; SWAP70; PTGES3; USP9Y; PDE4DIP; BAZ1A; PLRG1; CWC15; TUBB2A; RNF169; SUB1; H2AC21; GAPDHS; ANP32E; CHMP4B; ANP32B; CRYAB; SLC25A6
	Nuclear Chromosome	2×10^{-3}	CFDP1; ANP32E; BAZ1A
	Polymeric Cytoskeletal Fiber	9×10^{-3}	TUBB2A; TUBB; IQGAP2; CLASP2
COMPARTMENTS _Curated_2025	Microtubule	2×10^{-4}	TUBB2A; TUBB; CHMP4B; IQGAP2; CHMP5; CLASP2
	Nuclear Lumen	2×10^{-3}	YAP1; PDCL3; RNF169; FAHD1; SUB1; SRSF1; ANP32E; PLRG1; CWC15
COMPARTMENTS _Experimental_ 2025	Endoplasmic Reticulum	8×10^{-4}	RCN1; CALU; RRBP1; BAZ1A
	Cytoplasm	2×10^{-3}	PDCL3; RCN1; FAHD1; PTGES3; AKR1A1; CALU; PDCL; RRBP1; CRYAB
Jensen_ DISEASES_ Curated_2025	Eye Disease	1×10^{-2}	YAP1; IL1RN; CHMP4B; CRYAB; PQBP1
	Nervous System Disease	4×10^{-2}	YAP1; IL1RN; CFDP1; TRAPPC10; AP1S1; CHMP4B; A2M; CRYAB; PQBP1
	Disease of Metabolism	5×10^{-2}	CPS1; GLDC; CALU; A2M; COQ6

*GO enrichment analysis of 49 of 59 proteins enriched in cry-V96-treated hRPE cells under tunicamycin-induced ER stress for the most significant, relevant pathways for the following GO categories; Molecular Function, Biological Process, Cellular Component, Compartments, and Jensen DISEASES.
