# Supplementary material for: αB-crystallin elastin-like polypeptides for sustained ocular drug delivery
Source: Drug Deliv Transl Res. Author manuscript; Available in PMC 2026 Oct 6. (PMC13641684; doi:10.1007/s13346-026-02210-w)
Supplement: Supplementary file 2 [file NIHMS2211083-supplement-Supplemental_B.pdf]

Supplemental Appendix B: Raw Images of Gels and Western Blots

αB-crystallin elastin-like polypeptides for sustained ocular drug delivery

Sara Aly Attia[1], Amanda M. Tse[1], Ashley D. Ramirez[1], Maryam Izadiar[1], Ying Liu[2], Hao Guo[1], Angela Albanese[1], Whitaker Cohn[3], Adrienne W. MacKay[4], Paul M. Seidler[1], Ram Kannan[5,6], Parameswaran G. Sreekumar[5*], John Andrew MacKay[1,2,7 *]

[1] Department of Pharmacology and Pharmaceutical Sciences, University of Southern California Mann School of Pharmacy and Pharmaceutical Sciences, Los Angeles, CA 90033, USA

[2] Department of Ophthalmology, Roski Eye Institute, University of Southern California Keck School of Medicine, Los Angeles, CA 90033, USA

[3] Titus Family Department of Clinical Pharmacy, University of Southern California Mann School of Pharmacy and Pharmaceutical Sciences, Los Angeles, CA 90033, USA

[4] Department of Population and Public Health Sciences, University of Southern California Keck School of Medicine, Los Angeles, CA 90033, USA

[5] Doheny Eye Institute, Pasadena, CA 91103, USA

[6] Stein Eye Institute, University of California Geffen School of Medicine, Los Angeles, CA 90095, USA

[7] Alfred E. Mann Department of Biomedical Engineering, University of Southern California Viterbi School of Engineering, Los Angeles, CA 90089, USA

*co-corresponding authors

John Andrew MacKay, jamackay@usc.edu

Parameswaran G. Sreekumar, sparameswaran@doheny.org

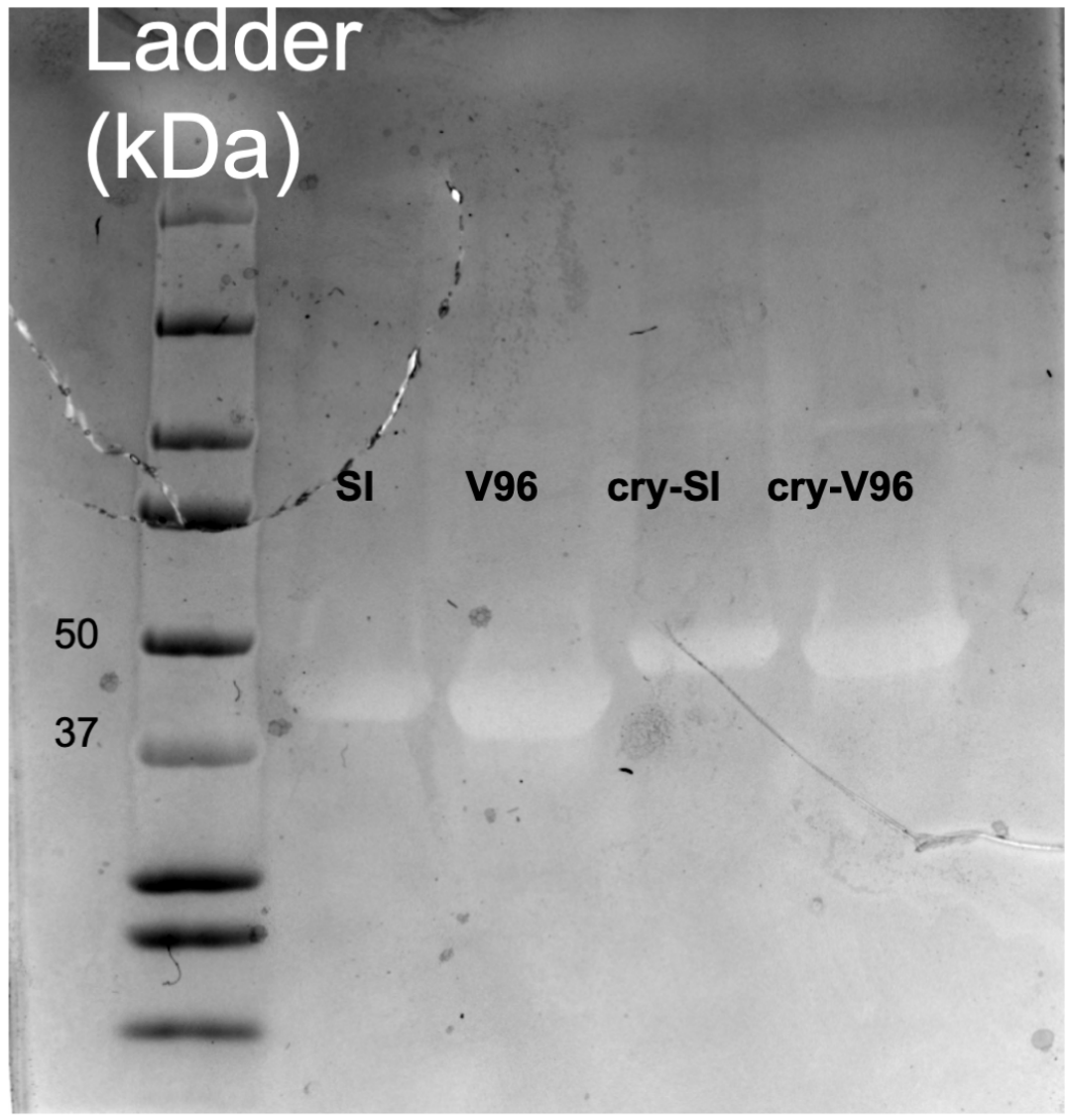

Supplementary Image S1. The uncropped gel corresponding to **Fig. 1**.

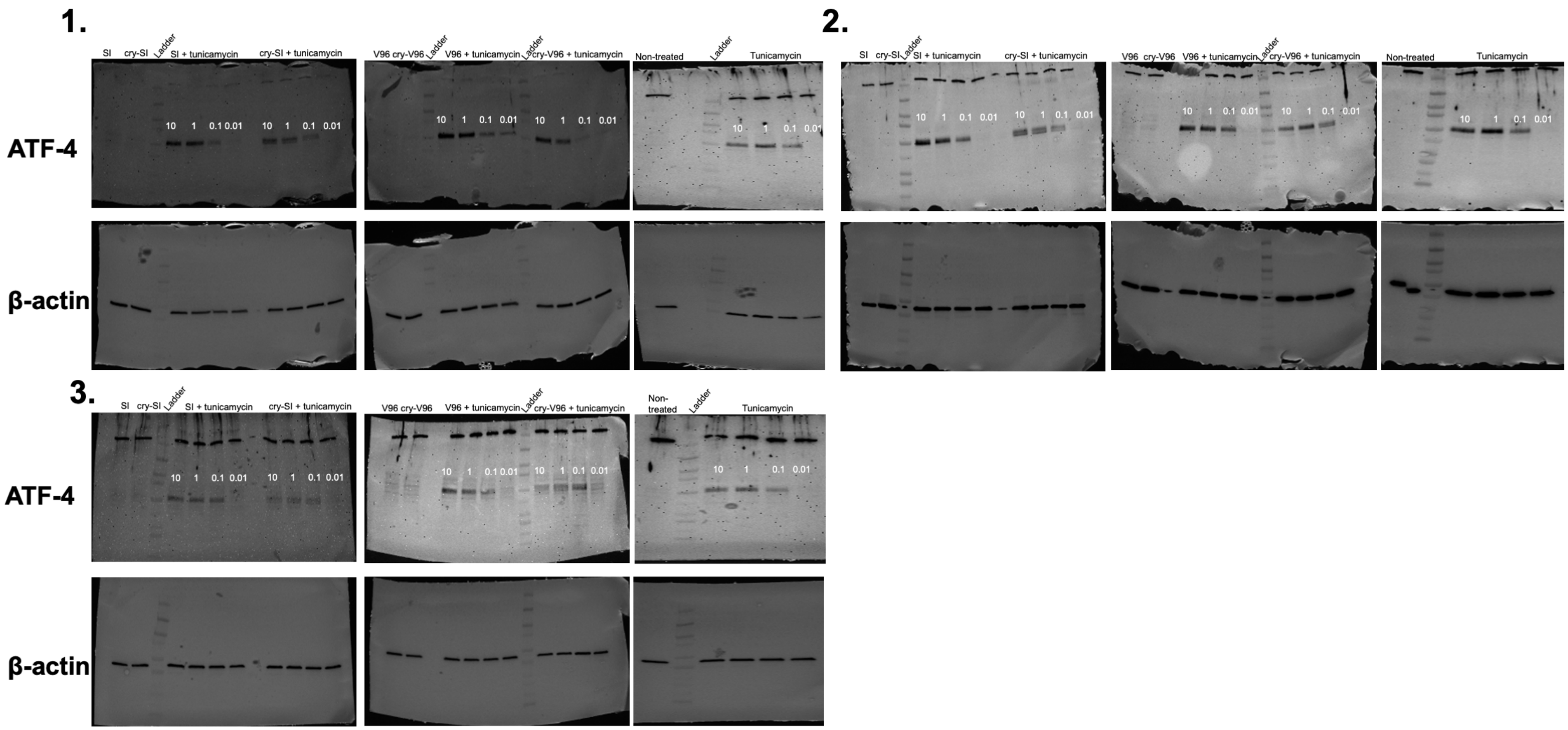

Supplementary Image S2. The individual Western whole gel blots corresponding to **Fig. 4A**, used to quantify data in **Figs. 4B,C**.

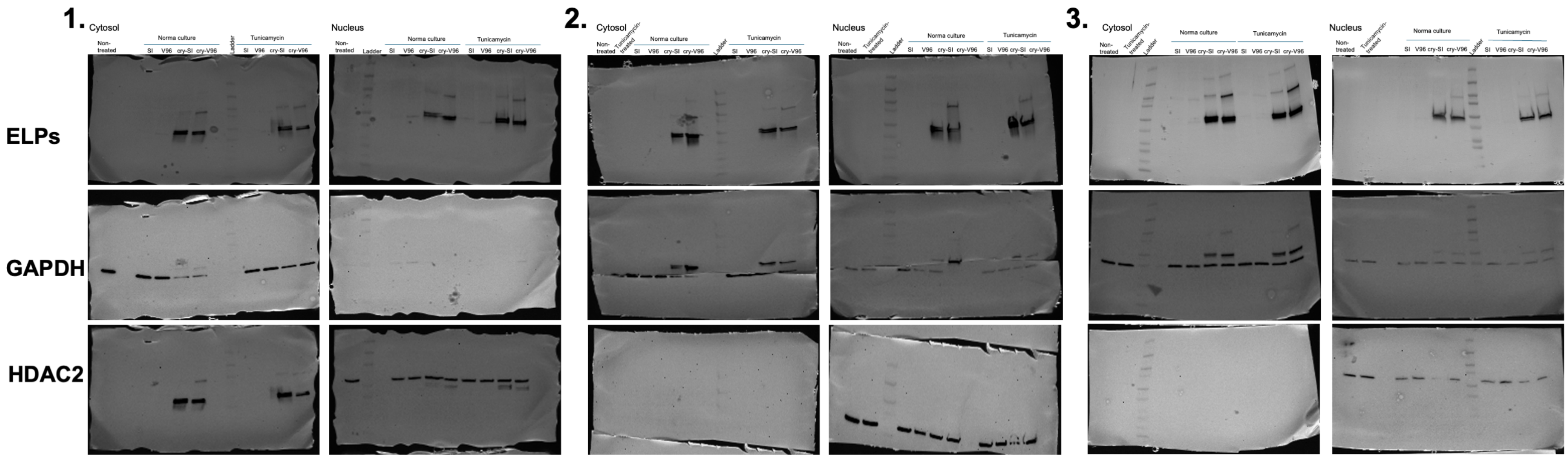

Supplementary Image S3. The individual Western whole gel blots corresponding to **Fig. 5A**, used to quantify data in **Fig. 5B**.

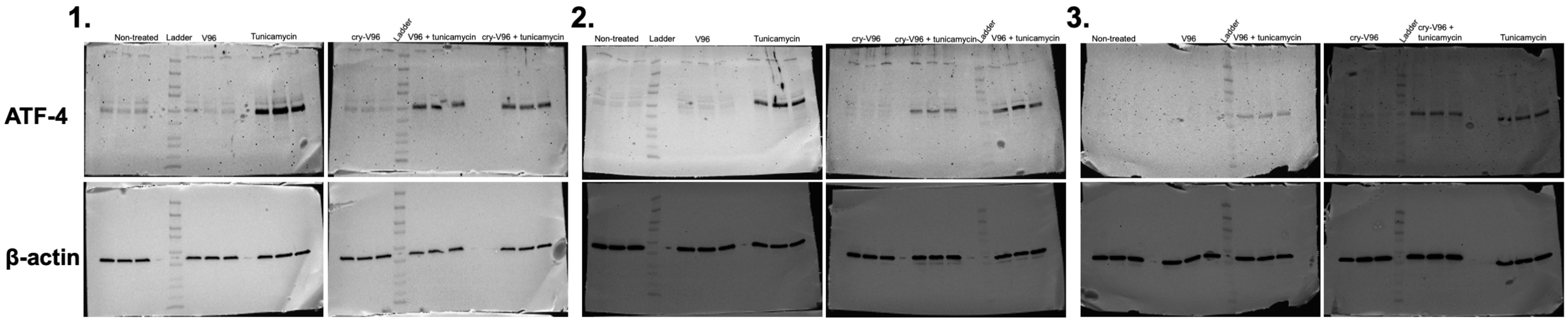

Supplementary Image S4. The individual Western whole gel blots corresponding to **Fig. 6A**, used to quantify data in **Fig. 6B**.

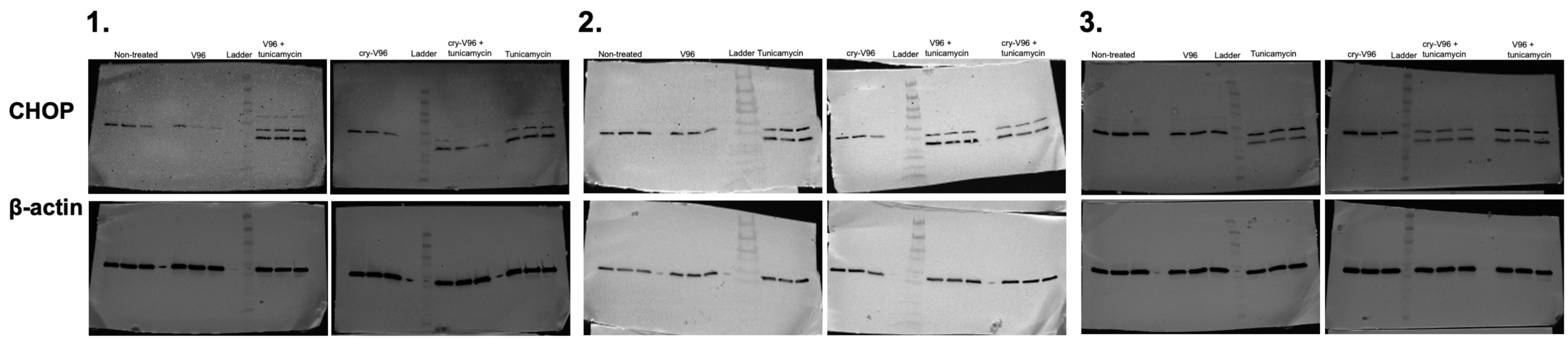

Supplementary Image S5. The individual Western whole gel blots corresponding to **Fig. 6C**, used to quantify data in **Fig. 6D**.

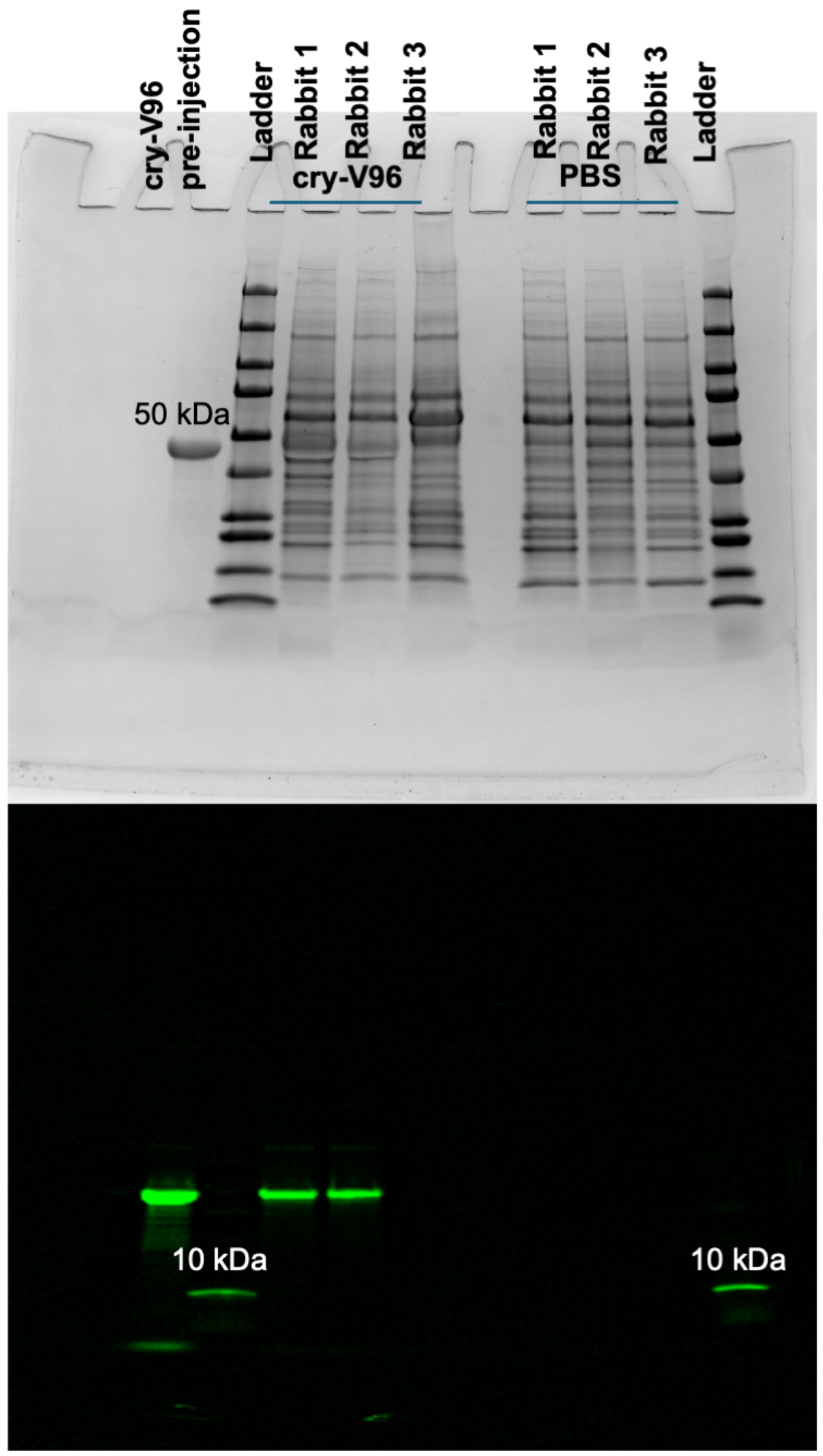

Supplementary Image S6. The uncropped gel corresponding to **Fig. 7E**.
